# Prediction of South American Leaf Blight and Disease-Induced Photosynthetic Changes in Rubber Tree, Using Machine Learning Techniques on Leaf Hyperspectral Reflectance

**DOI:** 10.3390/plants11030329

**Published:** 2022-01-26

**Authors:** Armando Sterling, Julio A. Di Rienzo

**Affiliations:** 1 Phytopathology Laboratory, Instituto Amazónico de Investigaciones Científicas SINCHI-Facultad de Ciencias Básicas, Universidad de la Amazonía, Florencia 180001, Colombia; 2 InfoStat Transfer Center, Facultad de Ciencias Agropecuarias, Universidad Nacional de Córdoba, Córdoba 5016, Argentina; dirienzo@agro.unc.edu.ar

**Keywords:** *Hevea brasiliensis*, *Pseudocercospora ulei*, spectral reflectance, machine learning, disease detection, precision crop protection, photosynthesis prediction

## Abstract

The efficiency of visible and near-infrared (VIS/NIR) sensors and predictive modeling for detecting and classifying South American Leaf Blight (SALB) (*Pseudocercospora ulei*) in rubber trees (*Hevea brasiliensis*) has been poorly explored. Furthermore, the performance of VIS/NIR analysis combined with machine learning (ML) algorithms for predicting photosynthetic alterations caused by SALB is unknown. Therefore, this study aimed to detect and classify the SALB levels, as well as to predict, for the first time, disease-induced photosynthetic changes in rubber trees. Leaf hyperspectral reflectance combined with five ML techniques (random forest (RF), boosted regression tree (BRT), bagged classification and regression trees (BCART), artificial neural network (ANN), and support vector machine (SVM)) were used. The RF, ANN, and BCART models achieved the best performance for classifying the SALB levels on the training dataset (accuracies of 98.0 to 99.8%), with 10-fold cross-validation repeated five times, and test dataset (accuracies of 97.1 to 100%). The ANN and RF models were better at predicting leaf gas exchange-related traits such as net CO_2_ assimilation rate (*A*) and extrinsic water use efficiency (*WUEe*) in the training (R^2^ ranged from 0.97 to 0.99) and testing (R^2^ ranged from 0.96 to 0.99) phases. In comparison, lower performances (R^2^ ranged from 0.24 to 0.52) were evidenced for the photochemical traits. This research provides a basis for future designs of a remote monitoring system based on early detection and accurate diagnosis of biotic stress caused by SALB, which is fundamental for more effective rubber crop protection.

## 1. Introduction

Rubber cultivation of *Hevea brasiliensis* (Will. Ex Adr. De Juss) Muell.-Arg is the main source of natural rubber in the world [1]. Global production is concentrated in the Asian continent (91.2%) [2] despite the fact that the rubber tree comes from the Amazon region in South America [3]. Latin America only represents 2% of global production [2]. This low representation in rubber production is mainly limited by the principal phytosanitary problem of this crop, a foliar disease known as South American leaf blight, which is caused by the fungus *Pseudocercospora ulei* [4] that affects the physiology of the plant [5,6] and gradually reduces latex production (reduction from 20 to 75%) [7]. This disease is managed mainly by genetic control, and monitoring is carried out using classic measurement methods [8].

Conventional diagnosis and monitoring methods for crops diseases mainly include field visual inspection, laboratory tests, and non-invasive detection techniques [9]. In particular, field methods can be subjective, untimely, expensive, and poorly reproducible, and laboratory tests such as molecular tests are limited in terms of early detection, control, and management [9,10]. On the other hand, the use of modern technologies for remote detection of plant disease, called “radiodiagnosis” by Zhang et al. [11], has shown high efficiency, simplicity, accuracy, speed, reproducibility, and accessibility for detecting and differentiating between healthy and infected leaves [9,11,12,13].

Remote sensing applied to plant disease allows the acquisition of biophysical information from plants mainly related to infection and physiological changes caused by pathogens [10,11,14,15,16,17]. According to Zhang et al. [11], four biophysical changes can be detected: (1) reduction of biomass and decrease in leaf area index (LAI), (2) lesions from infection, (3) destruction of pigment systems, and (4) wilting.

Based on physical principles, remote sensing systems can be classified into three types: (1) visible and infrared spectral systems (VIS-NIR, 400–2500 nm), (2) Fluorescence and thermal systems, and (3) synthetic aperture radar (SAR) and light detection and ranging equipment (Lidar) systems [11]. In particular, VIS-NIR spectroscopy systems are based on the relationship between the “source” signal or irradiance (electromagnetic radiation) interacting with the surface, and the reflected, “received” signal at the sensor (surface-reflected energy), which is known as spectral reflectance [9,11].

The spectral characteristics of light reflected by the leaves can be an indicator of biotic stress in plants with different types of pathogens [9]. Thus, several studies have demonstrated the potential of spectral reflectance and VIS-NIR spectroscopy systems (wavelength: 400–1000 nm) in remote detection and discrimination of plant pathogens [10,18,19,20,21,22], as well as the use of hyperspectral data as a proxy to analyze physiological changes in plants from different types of stress [15,16,17,23,24]. In the VIS-NIR sensors, the most important regions of electromagnetic spectrum correspond to the visible portion of the spectrum (VIS, wavelength: 400–750 nm), mainly related to changes in chlorophyll and carotenoid contents, and the near-infrared (NIR), more precisely the short-wavelength region (SW-NIR, wavelength: 750–1300 nm), which is associated with structural discontinuities found in the leaf mesophyll [9,11]. However, the level at which different diseases cause various spectral changes in plants and the degree to which the spectral reflectance changes for a particular stress agent vary among species, plants, organs, and tissues [9,10,25]. Various spectral aspects related to biotic stress are still unresolved in many pathosystems because of the specificity of the host-pathogen interaction [12].

Additionally, spectral data require the use of advanced data analysis methods and calibration of specialized algorithms in accordance with the multidimensional nature of the spectral information [9,14]. Traditional data analysis techniques, such as regression models and linear discriminant analysis, have various assumptions that, in many cases, are not met because of the non-linear relationships between response and predictor variables, which limits the use of these techniques when modeling highly multidimensional data, such as spectral data [14,26]. In contrast, modern data-analysis techniques, such as machine learning (ML) algorithms adapt to any mixture of data types and can handle huge volume data. Thus, techniques such as the artificial neural network (ANN) and support vector machine (SVM) utilize a mechanism that transforms the input into the output through complex mathematics processes [26,27,28]. Furthermore, other techniques such as random forest (RF), boosted regression tree (BRT), and bagged classification and regression trees or bagged carts (BCART) utilize more advanced strategies to augment the model’s performance through a process known as ensemble or meta-learning (i.e., combining multiple weaker learners to create a stronger learner) [14,26,28]. These techniques have demonstrated a high performance for predicting different diseases and disease-induced physiological changes in plants from spectral data [13,14,16,19,29,30,31,32]. However, the use of ML algorithms for predicting disease levels, as well as physiological changes caused by disease in the same host, is poorly documented.

In the *H. brasiliensis*-SALB pathosystem, Sterling and Melgarejo [20] used spectral vegetation indices (SVIs) and multiple linear regression to analyze foliar spectral changes caused by the fungus *P. ulei*, and Lucas Domingos da Silva et al. [33] used near-infrared spectroscopy (NIRS) technique and PLS-DA (partial least squares discriminate analysis) modeling for the tree leaves classification of mature leaves with and without SALB.

Despite the efficiency of VIS/NIR sensors and predictive modeling for the detection and classification of leaves with and without SALB symptoms, its potential for predicting disease levels has been poorly explored. Furthermore, the performance of VIS/NIR sensors and ML algorithms for predicting photochemical and non-photochemical photosynthetic alterations caused by SALB has not been documented. The development of predictive models based on spectral reflectance and ML techniques is important because it will not only allow detection and discrimination of the intensity of the symptoms generated by SALB, but also to detect and predict the biotic stress generated by this disease. This information is essential for the early diagnosis and effective management of SALB, as well as for the use of appropriate management practices to improve the physiological status and productive performance of rubber trees affected by this disease. The hypothesis of this study was that the detection and classification of SALB levels in young leaves of *H. brasiliensis*, as well as the prediction of photochemical and non-photochemical photosynthetic changes caused by this disease, can be captured by VIS/NIR analysis combined with ML algorithms.

Therefore, this study had the following objectives: (1) to analyze changes in leaf hyperspectral reflectance caused by SALB using VIS-NIR sensors; (2) to select wavelengths with sufficient ability to discriminate between levels of SALB; and (3) to develop models based on different machine learning techniques to classify SALB levels and predict disease-induced photochemical and non-photochemical photosynthetic changes in rubber trees.

## 2. Materials and Methods

### 2.1. Experimental Conditions and Obtaining of Disease Severity Classes

**Figure 1:**
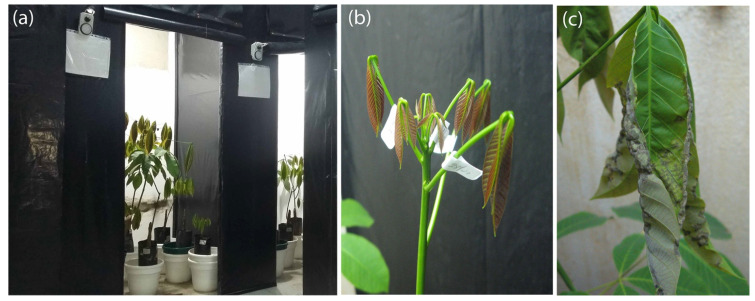
Experimental conditions of the study. (**a**) Conditions-controlled room with *Hevea brasiliensis* plants, (**b**) healthy leaflets (0) in stage B at 0 days after inoculation (dai), (**c**) diseased leaflets in stage C with SALB severity class ‘4’ at 20 dai.

This experiment was conducted in a climate-controlled room (an air temperature = 23 °C, a relative humidity range = 90–95% and a photoperiod of 12 h dark/12 h light at 2000 lux) at the Phytopathology Laboratory of the SINCHI Amazonian Institute of Scientific Research (Florencia, Caquetá, Colombia) (1°37′03″ N and 75°37′03″ W). Ten healthy 4-month-old rubber plants for the two cultivars with different susceptibility to SALB, FX 3864 (PB 86 × FB 38) (high susceptibility) [5,34,35] and FX 4098 (PB 86 × B 110) (low susceptibility) [5,35,36], were grown in individual plastic bags with 7 kg of Caquetá soil [37] and arranged in pots to perform the experiment (Figure 1a). The selected plants had stems that were 75–80 cm high, with a 2–2.5 cm girth, and second leaflets whorl in stage B [38] (i.e., reddish, green nerves visible and surface gleaming leaflets) (Figure 1b). The two tested cultivars were made by Ford Plantations in Brazil from 1935 to 1943 [8] and were commercially introduced to Colombia in 1964 and 2000, respectively [39]. In this study, these two clones were used to obtain an infection gradient and achieve the lowest and greatest SALB severity classes [20].

The plants of each cultivar were divided into two groups (each with five plants): without inoculation and with inoculation (Table 1). Thus, four rubber leaflets per plant were inoculated with a highly aggressive 17i VIF isolate of *P. ulei* fungus (SALB) [40], belonging to the isolates bank of the SINCHI Phytopathology Laboratory, following the methodology by Sterling and Melgarejo [5]. 10-day-old leaflets in stage B (Figure 1b) were sprayed on the abaxial surface with a suspension containing 2 × 10^5^ conidia mL^−1^.

After inoculation, the plants were kept in darkness for 24 h and, thereafter, were subjected to a photoperiod of 12 h dark/12 h light at 2000 lux until day 20 in a conditions-controlled room. The disease severity was monitored at 0, 4, 8, 12, 16, and 20 days after inoculation (dai) to obtain different SALB levels in young leaf stages (leaflets B and C).

**Figure 2:**
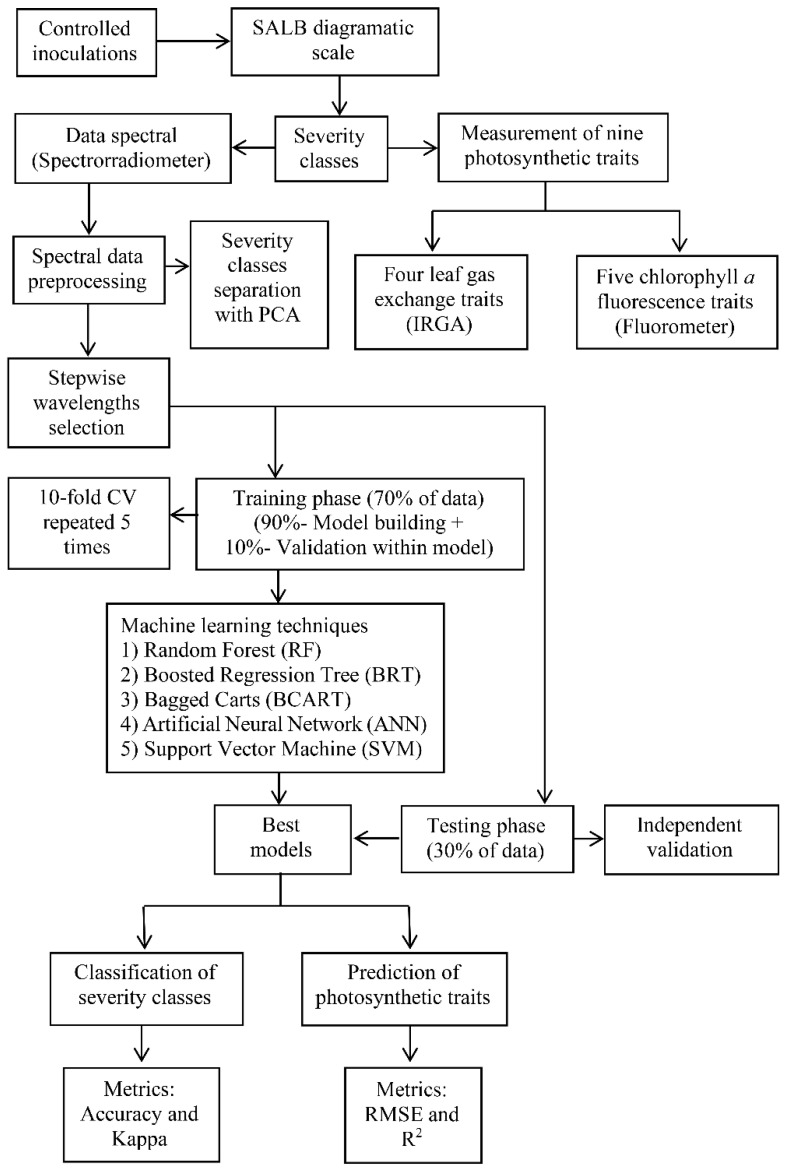
Flowchart of the general methodology followed in the research.

When SALB visible lesions were recognized (Figure 1c) and classified according to Gasparotto et al.’s [7] severity scale adapted by Sterling and Melgarejo [41] (i.e., the percentage of foliar area with disease symptoms and signs), five treatments (levels or classes of SALB severity) were established in a completely randomized experimental design. Thus, the plants that had the same severity in each day of sampling and were assigned in each severity class (Table 1). Figure 2 (flowchart) shows the overall methodology followed in this study, which begins with obtaining the SALB severity classes and nine photosynthetic traits, continues with capturing and preprocessing the spectral signatures and selection of optimal spectral bands and culminates with the prediction of the SALB levels and disease-induced photosynthetic changes using five machine learning techniques.

### 2.2. Photosynthetic and Spectral Data Acquisition

Photosynthetic and spectral measurements were performed on the most severely attacked leaflet per plant in each severity class in each day of sampling (0, 4, 8, 12, 16, and 20 days) (Table 1). Nine photosynthetic traits related to biotic stress caused by SALB in *H. brasiliensis* [5,6] were measured following the methodology of Sterling and Melgarejo [5]: (1) four-leaf gas exchange traits, net CO_2_ assimilation rate (*A*) (μmol CO_2_ m^−2^ s^−1^), transpiration rate (*E*) (mmol H_2_O m^−2^ s^−1^), stomatal conductance (*g_s_*) (mol H_2_O m^−2^ s^−1^), and extrinsic water use efficiency (*WUEe*) (μmol CO_2_ mmol^−1^ H_2_O), using an IRGA portable infrared gas analyzer (TPS-2 Portable Photosynthesis System, USA); and (2) five chlorophyll *a* fluorescence traits in leaflets adapted to darkness using dark-adaptation leaf-clips for 30 min, maximum photochemical efficiency of PSII (*F_v_*/*F_m_*), efficiency in capturing excitation energy for the open reaction centers of the PSII (*F_v_′*/*F_m_′*), ETR (electron transport rate through PSII), non-photochemical quenching of PSII (NPQ), and coefficient of photochemical quenching (*qP*), using a field-portable pulse-modulated chlorophyll fluorometer (Hansatech Instruments, Hoddesdon, England).

After the photosynthetic traits’ measurements, the spectral reflectance was measured using an Apogee PS-100 spectroradiometer (spectral range of 350 to 1000 nm) and SpectraWIZ operation software (StellarNet Inc., Tampa, FL, USA) according to the methodology by Sterling and Melgarejo [20]. Thus, each spectral reading was automatically averaged with 30 spectral reflectance, using a spectral interval of 1 nm. Before the spectral readings, the spectroradiometer was calibrated with a white surface of maximum reflectance using a 100-W halogen lamp to illuminate the surface and another black surface of minimum reflectance. The spectral range between 400 and 900 nm (including 501 spectral bands) were used. The bands below the 400 nm and above the 900 nm were not considered because of the noise, scattering, or deformed spectra (readings error). Similar spectral intervals have been considered in various biotic stress studies because of the alterations caused by the pathogens in the photosynthetic pigments and in the mesophyll structure, also affecting the leaf spectral reflectance properties in the VIS and NIR regions, respectively [15,20,21,42].

Three reflectance spectra of the adaxial face of each selected leaflet were measured and then averaged per plant (spectral sample). Because of the design, 120 spectral samples distributed in the five severity classes, as shown in Table 1, were collected during six days of sampling.

### 2.3. Spectral Data Pre-Processing

Two spectral preprocessing methods [43,44,45] were carried out on the reflectance spectra data, using ParLes [43]: (1) multiplicative scatter correction (MSC) (i.e., correction of the scatter effect), and (2) smoothing/denoising with Savitzky–Golay filter (i.e., reduction of random noise with a second polynomial order). Then, the preprocessed data were imported to InfoStat v. 2020 [46] to plot average reflectance curves from each severity class.

### 2.4. Photosynthetic Data Preparation

The means and standard error values of the dataset based on nine photosynthetic traits were estimated for each SALB severity class in both leaf stages (leaflets B and C) to analyze the data variation. The descriptive statistics were performed in InfoStat v. 2020.

### 2.5. Severity Classes Separation Using Principal Component Analysis (PCA)

A principal component analysis (PCA) was used to visualize the separation of SALB severity classes in a two-dimensional graphic (PC1 and PC2, which capture the maximum variance) based on leaf hyperspectral reflectance. This technique has been used in previous works to analyze changes in the variance of the spectral reflectance among different levels of categoric variables to identify how well these categories can be separated [21,45,47,48]. The factor loading plot of each PCA, was also analyzed, defined as the importance of each variable (wavelength) on each principal component, to identify spectra regions that have a higher capacity for discriminate among classes [47,49]. The PCA was performed in InfoStat v. 2020.

### 2.6. Stepwise Wavelengths Selection

Prior to the machine learning model’s tuning, a stepwise selection (or sequential replacement) was carried out with all spectral samples (*n* = 120) to reduce the dimensionality of the predictors (501 wavelengths obtained by reflectance data), using InfoStat v. 2020 at a *p*-value < 0.05. This technique selects the wavelengths that best explain the differences between the severity classes, avoiding variable variance inflation and removing the multicollinearity between wavelengths [21,42,48]. Stepwise selection is a combination of forward (add the most contributive predictors) and backward (remove the least contributive predictors) strategies that select and retain the variables that are more important and maximize the coefficient of determination (R^2^), which can be applied in the high-dimensional configuration, where the number of samples n is inferior to the number of predictors *p*, such as spectral reflectance and genomic data [50,51,52].

### 2.7. Data Splitting Strategy

After the stepwise wavelength selection for the best SALB severity class differentiation, the spectral samples were divided into two parts, 70% of which were used for training and 30% for testing. This was performed with a stratified random sampling (strata: severity class) in R language, v. 4.0.3 [53] and the interface in RStudio v. 1.3.1093 [54] using the function *createDataPartition* from the package caret [55].

### 2.8. Machine Learning Techniques

Five machine learning techniques (learning task dual: classification and numeric prediction) were applied for classifying the severity classes and predicting the nine photosynthetic traits: random forest (RF), boosted regression tree (BRT), bagged classification, and regression trees or bagged carts (BCART), artificial neural network (ANN) and support vector machine (SVM) [26,28,56,57]. These models were fitted and validated using different tuning parameters in R language with the *train* function from the package caret [55]. A grid-Search was used to explore different values of the hyperparameters of the RF, BRT, ANN, and SVM algorithms using the tuneGrid argument of the *train* function. No hyperparameter search was required for BCART [26,55]. A short overview of each individual machine learning technique was provided.

#### 2.8.1. Random Forest (RF)

The RF algorithm is a non-linear ensemble technique focused only on decision trees for classification or regression [26,28,32]. This technique combines the principles of bagging with random (bootstrap) feature selection to aggregate versatility and power in a single machine learning, which makes it possible to process extremely large datasets [28]. Recent studies have demonstrated the effectiveness of RF for discriminating hyperspectral data in remote sensing [16,29,30,31,47]. In this study, the train model ‘rf’ was used, and tuning parameters are shown in Table S1 (Supplementary Materials).

#### 2.8.2. Boosted Regression Tree (BRT)

Boosting models were initially developed for classification (boosted classification) and were later used for regression fitting (boosted regression). Therefore, the BRT algorithm integrated two statistical algorithms: CART and boosting algorithm [26,58]. BRT is a powerful ensemble technique that avoids overfitting and boosts the performance of weak learners to obtain the performance of stronger learners [28,30]. Previous studies have confirmed the high performance of BRT from hyperspectral data [13,30,31,47]. The train model ‘gbm’ and tuning parameters are shown in Table S1 (Supplementary Materials).

#### 2.8.3. Bagged Carts (BCART)

Bagging, or bootstrap aggregation, is one of the first ensemble techniques that uses bootstrapping for classification or regression fitting to construct and ensemble [26,28,59]. The model predictions are combined by voting (for classification) or averaging (for numeric prediction) [28]. This technique can perform quite well with unstable learners as the CART algorithm, which is known as bagged trees or bagged carts (BCART) [26,28]. Previous studies have confirmed the performance of bagged trees in modeling using machine learning and remote sensing [57,60,61]. The train model ‘treebag’ and tuning parameters are shown in Table S1 (Supplementary Materials).

#### 2.8.4. Artificial Neural Network (ANN)

The ANN algorithm is a powerful nonlinear regression technique, just like a brain uses a network of neurons (relationship between inputs signals and output signal) to provide great learning ability [26,28]. ANN employs artificial network nodes (neurons) to solve complex and hard learning problems [28]. Various recent studies have demonstrated the power of this technique for classifying or predicting different biological phenomena from hyperspectral data [13,16,19,47,57]. The train model ‘nnet’ and tuning parameters are shown in Table S1 (Supplementary Materials).

#### 2.8.5. Support Vector Machine (SVM)

The SVM algorithm is an extremely powerful technique that creates a flat boundary called a hyperplane, which combines the abilities of both the nearest neighbors learning, and the linear regression modeling to model highly complex problems [26,28]. The nonlinear relationships between variables are modeled using a process known as the kernel trick (kernel functions) [28]. Several studies have shown the high capacity of these algorithms for solving complex real-world problems from hyperspectral data [13,16,29,47,57]. A SVM was tested with nonlinear kernel (radial basis function) [26,55]. The train model ‘svmRadial’ and tuning parameters are shown in Table S1 (Supplementary Materials).

### 2.9. Models Performance Evaluation

The model performance was evaluated twice: first in the training phase (70% of data) using repeated k-fold cross-validation (i.e., 90% of data is used to build model and 10% is used for validation within the model), and second, using the independent or external validation dataset for testing phase (30% of data) [26,28,62].

Thus, a 10-fold cross-validation repeated five times (i.e., 10-fold CV 5 times) method in the R package caret (trainControl tuning parameter) was used to generate and select the best models from the training samples [26,28]. The model’s performance for classifying severity classes was carried out by comparing the accuracy and Kappa statistics, while the performance for predicting the nine photosynthetic traits compared the root-mean-square error (RMSE) and R^2^ statistics [26,28,57]. The resampling results of the models were collected into a single object and the mean values of accuracy and RMSE displayed in dot plots using function *resamples* from the R package caret [55]. All models were analyzed with pair-wise comparison (metric: accuracy or RMSE) using *t*-tests with Bonferroni correction (confidence level = 0.95) [26,63] and the *diff* function in the R package caret [55].

The future performance of the best models on unseen data (i.e., test dataset) was evaluated from predicted values obtained with *predict* function from R package caret and two statistical procedures: (1) statistics by class (metrics: sensitivity, specificity, positive predictive value (PPV), negative predictive value (NPV) and balanced accuracy (BC)) using the *confusionMatrix* function from the R package caret [55] for classifying the severity classes [26,28,47]; and (2) linear regressions with diagnostic scatterplots using the *lm* function from R package stats [53], comparing measured versus predicted values (metrics: RMSE and R^2^) for predicting the photosynthetic traits [16,26,52,62].

## 3. Results

### 3.1. Photosynthetic Traits

The means of the photochemical and non-photochemical foliar photosynthetic traits were lower as the SALB severity increased, with the exception of NPQ, which had higher means at the higher severities (Table 2). In general, these changes were more intense in the C leaflets than in the B leaflets, mainly in the gas exchange traits. The B leaflets had a maximum severity of ‘3’, and the mean of *A* was reduced by 176.10% with respect to the healthy leaflets (‘0’). The C leaflets had a maximum severity of ‘4’, with a reduction of 196.04%.

### 3.2. Leaf Reflectance Spectra

**Figure 3:**
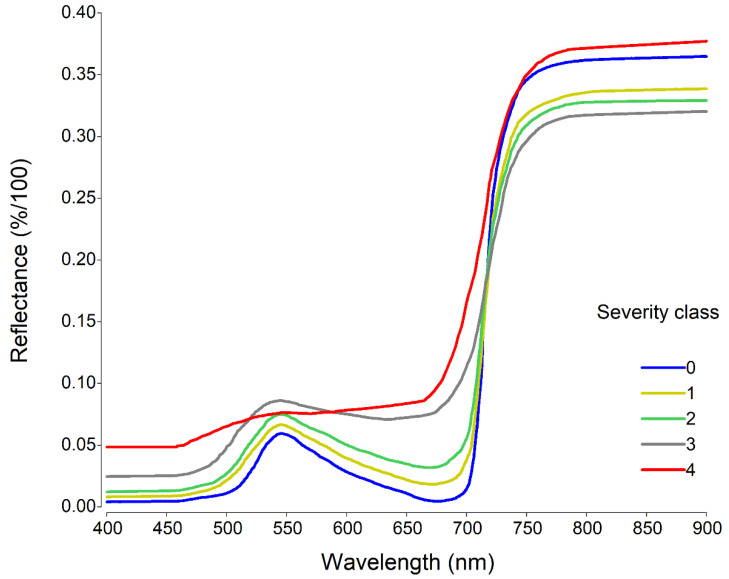
Spectral reflectance signatures of healthy leaflets (0) and SALB severity classes. Classes ‘0’, ‘1’, ‘2’, and ‘3’ corresponding to the mean of leaflets in stages B and C, and class ‘4’ to leaflets in stage C.

Figure 3 shows the spectral reflectance curves of the different classes of SALB severity. The spectral reflectance in the NIR region (750–900 nm) had higher values than the VIS region (400–750 nm) in all severity classes. In the SIV region, reflectance increased with increasing severity, reaching a peak greater than 545 nm with severity class ‘3’. On the contrary, in the NIR region, the reflectance was lower with the higher severity, with the exception of severity class ‘4’, which had higher reflectance values in the VIS and NIR regions.

### 3.3. Separability of Severity Classes

**Figure 4:**
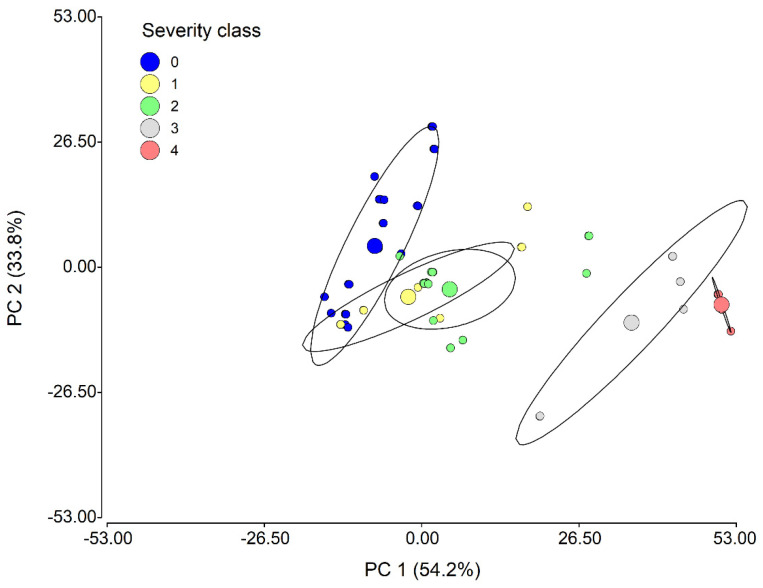
SALB severity class separation using principal component analysis (PCA). The circle represents the 95% confidence ellipses and the major points represent the centroids of each class. PC 1 and PC 2 (Principal component 1and 2, respectively).

The PCA showed that the first two components (PC1 and PC2) captured 88% of the total variability, where PC1 explained 54.2% of the variability, and PC2 explained 33.8% of the remaining variability (Figure 4). An appreciable separation was observed between the different SALB severity classes, mainly between class ‘0’ (healthy leaflets) and the higher severity classes (‘3’ and ‘4’). Classes ‘2’ and ‘3’ presented a partial overlap in the center of the arrangement plane, with positions closer to class ‘0’.

**Figure 5:**
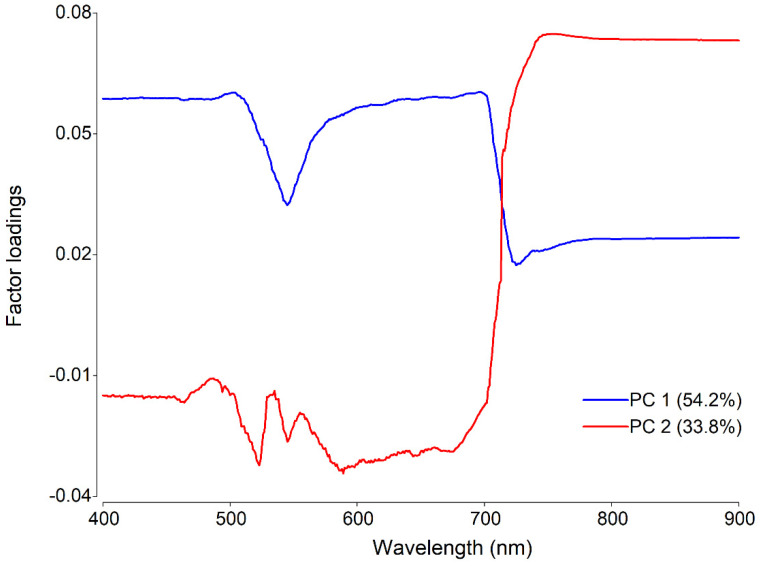
Factor loadings plots of the PCA (PC1, blue curve, and PC2, red curve) under different SALB severity classes.

The factor loading analysis from the PCA made it possible to determine the most important regions of the spectrum associated with each principal component (Figure 5). In the VIS region, the largest eigenvectors associated with PC1 were observed, while the NIR region had the largest eigenvectors associated with PC2. In the VIS region, the most important regions were 495 at 510 nm and 686 at 705 nm, while in the NIR region, the highest eigenvectors were obtained in the range from 750 to 775 nm.

### 3.4. Selected Stepwise Wavelengths

Of the 501 spectral bands obtained from the VIS/NIR spectrum, the stepwise procedure selected 20 wavelengths that had the best ability to discriminate the different classes of SALB severity (adjusted R^2^ = 0.98; RMSE = 0.028; *p* < 0.001). Among the retained wavelengths: (1) 19 corresponded to the VIS region, with 9 in the green region (524, 525, 533, 537, 549, 551, 560, 561, and 565 nm), 3 in the yellow/orange region (582, 596 and 602 nm), and 7 in the red region (630, 680, 697, 699, 703, 707, and 709 nm); and (2) 1 associated with the NIR region (755 nm).

### 3.5. Classification of Severity Classes

The results of the five machine learning techniques using the 20 selected wavelengths are shown in Table 3. A higher performance in the training phase (86 samples) with the 10-fold CV 5 times method was evidenced in the RF, ANN, and BCART models, which had the highest values of accuracy (99.8, 98.1, and 98.0%, respectively) (Figure S1a; Supplementary Materials) and Kappa coefficient (0.99, 0.97 and 0.97, respectively). The pair-wise comparison with *t*-tests and Bonferroni correction based on accuracy values showed significant differences in RF vs. BRT (*p* = 0.001), RF vs. SVM (*p* = 0.021), and BCART vs. BRT (*p* = 0.006). No significant differences were found between RF, ANN, and BCART (*p* > 0.05).

Conversely, in the testing phase (34 samples) with the independent validation method, the highest values of accuracy (100.0%) and Kappa (1.00) were obtained in the ANN and SVM models (Table 4). In addition, five parameters of the confusion matrix were used to analyze the performance of the five models (Table 4). It was evidenced that the ANN and SVM models had the highest sensitivity, specificity, positive predictive value (PPV), negative predictive value (NPV), and balance accuracy in all SALB severity classes. Lower values of these parameters were obtained for severity classes ‘1’ and ‘2’ in the RF, BRT, and BCART models. Class ‘1’ had the lowest values of sensitivity, NPV, and balance accuracy for these three models, while class ‘2’ had the lowest values of specificity and PPV. The lower performance observed in those models was congruent with the overlapping evidence between the classes ‘1’ and ‘2’ in the PCA (Figure 4). Overall, the five models had a perfect balance accuracy in healthy leaflets (class ‘0’), and the ANN, SVM, RF, and BCART models had the same performance in the highest classes (‘3’ and ‘4’).

### 3.6. Prediction of Photosynthetic Traits

Table 5 shows the results of the performance of the five machine learning techniques for the nine photosynthetic traits in the training (i.e., with the 10-fold CV 5 times method; Figure S1b–j in Supplementary Materials) and testing phases (i.e., with the independent validation method; Figure S2 in Supplementary Materials).

Overall, the five models had a higher performance for predicting leaf gas exchange traits than chlorophyll-a fluorescence traits. The *A* and *WUEe* traits had the most accurate predicted values in the different models, while *F_v_′*/*F_m_′* and NPQ had the opposite result. Nevertheless, the predictions were significant, where 91.11% were highly significant (Figure S2). The RF, SVM, and BRT models had a higher performance in the training phase, while, in the testing phase, the RF model was the best. The highest performance in both phases for the same technique was evidenced in SVM for *E*, RF for *ETR*, and RF for *qP* (Table 5).

In the training phase, the ANN model had the highest performance for *A*; the SVM model for *E* and *g_s_*; the BRT model for *WUE_e_* and NPQ; and the RF model for *F_v_*/*F_m_*, *F_v_′*/*F_m_′*, ETR, and *qP* (Table 5). In addition, the pair-wise comparison with T-tests and Bonferroni correction based on RMSE values showed no significant differences (*p* > 0.05) in ANN vs. BRT for *A*; SVM vs. RF and BRT for *E*; SVM vs. RF for *g_s_*; BRT vs. ANN and SVM for *WUEe*; RF vs. other models for *F_v_*/*F_m_*; RF vs. BRT, BCART and SVM for *F_v_′*/*F_m_′*; BRT vs. RF, ANN and SVM for NPQ; RF vs. BRT and SVM for ETR; and RF vs. other models for *qP*.

In the testing phase, the RF model had the highest performance for *A*, *WUEe*, *g_s_*, NPQ, ETR, and *qP*; the SVM model for *E*; the BCART model for *F_v_*/*F_m_*; and the ANN model for *F_v_′*/*F_m_′*.

## 4. Discussion

### 4.1. Changes in Leaf Spectral Reflectance

The spectral response observed in the present study was similar to that reported in various pathosystems, including *H. brasiliensis*-*P. ulei* [10,19,20,21,33,45]. The first symptoms of SALB include the appearance of necrotic or chlorotic lesions [41] that directly affect the spectral reflectance in the VIS region (400–700 nm) [20], altering the concentration of photosynthetic pigments such as chlorophyll-*a*, *b*, and carotenoids, which leads to an increase in VIS reflectance as the severity of the disease increases [11,21,45,64]. The increase in SALB severity generated physiological alterations in *H. brasiliensis*, which reduced the photosynthetic efficiency of the plants (Table 2), similar to previous studies [5,6,35].

In comparison, the variation in spectral reflectance in the NIR region (750–1300 nm) was mainly associated with the mesophilic leaf structure since the cellular damage caused by pathogens produces a strong dispersion of electromagnetic energy at the mesophyll level, which causes reflectance to decrease when disease severity increases [18,21,45,47]. However, in the present study, severity class ‘4’ presented the highest reflectance in the NIR region, probably influenced by the greater reflectance in the stage C leaflets with high susceptibility (FX 3864) as compared to low susceptibility clone (FX 4098) [20], since severity ‘4’ was only observed in the C leaflets in clone FX 3864 (Table 1).

Similar results were reported by Furlanetto et al. [21], who observed a strong increase in reflectance in the VIS in the region between 500 and 700 nm when the severity of Asian soybean rust increased and lower reflectance at higher severity in the NIR region (750–1000 nm). Zhao et al. [45] showed how increasing the severity of wheat powdery mildew produced an increase in reflectance in the range between 460 and 710 nm, contrary to the reflectance observed within the spectral range of 730 to 900 nm.

Our results confirmed the capacity of remote sensing methods based on hyperspectral reflectance in the VIS/NIR region for detection plant diseases and discriminating between damage levels [9,10,11,25], which in *Hevea* represents a promising tool for the early detection and discrimination of SALB in relation to other diseases, such as anthracnose (*Colletotrichum* spp.) and black crust (*Phyllachora huberi*), which are closely associated with SALB symptoms at the leaf level [7,65].

### 4.2. Optimum Spectral Bands

The separation of SALB severity classes obtained with PCA (Figure 4) showed a greater overlap between classes ‘1’ and ‘2’ (i.e., in both classes, some points moved away from their centroid and were found together). This was mainly due to less visual differentiation in the proportion of diseased leaf surface, making the reflectance pattern similar [20]. In contrast, the other classes were better discriminated and separated in the PCA plot.

However, the separation of the SALB levels observed in our study was similar to that reported in previous studies for other diseases, such as Asian soybean rust [21] and wheat powdery mildew [45]. Thus, the PCA eigenvector matrix determined that the highest variance captured by PC1 (54.2%) was related to changes in the spectral response of *H. brasliensis* as evidenced in the VIS region, while PC2 (33.8%) was mainly associated with reflectance in the NIR region. According to Furlaneto et al. [21], this spectral variability captured by PC1 and PC2 was related to changes in the concentration of the photosynthetic pigments and in the internal structure of the leaf caused by the pathogen, respectively.

Although the PCA showed wavelengths with a high contribution in PC1 and PC2 for the VIS and NIR regions, respectively, the stepwise selection identified 20 statistically optimal spectral bands for discriminating SALB severity classes, especially in the VIS region, which indicated that the greatest impact from SALB on the spectral response was associated with the green, yellow/orange and red regions, which are strongly related to chlorophylls *a* and *b* [20,64,66].

Similar results were reported by Furlaneto et al. [21], who identified 87 wavelengths with the stepwise procedure that were statistically better at discriminating different levels of Asian rust soybean, 27 bands in the VIS region and 60 in the NIR region. Zhao et al. [45] identified 12 sensitive bands with PCA to discriminate different levels of wheat powder and mildew (492.7, 551.5, 665.2, 675.8, 713.4, 749.1, 750.5, 769.6, 778.2, 783.5, 808.6, and 853.6 nm). Marín-Ortíz et al. [15] reported five specific spectral bands that are highly correlated with increases in *F. oxysporum* in roots and leaves of tomato plants: two in the VIS range (448–523 nm and 624–696 nm) and three in the NIR region (740–960 nm, 973–976 nm, and 992–995 nm).

### 4.3. Classification of SALB Levels

Several studies have confirmed the potential of the combined use of spectral reflectance and machine learning (ML) algorithms for detecting various diseases in plants [9,11,14,25], and others have used reflectance to predict some morphophysiological traits [13,23,29,47]. However, there are few studies that have integrated reflectance, disease, physiology, and ML algorithms [11,12], and no study has used this approach for SALB in rubber trees. Mahlein [12] confirmed that the interaction of biotic and abiotic stresses, sensor development, informatics, and ML must be linked to achieving a highly interdisciplinary approach for improving plant health management.

Deng et al. [22] tested six ML algorithms (logistic regression, decision tree, support vector machine (SVM), K-nearest neighbor (KNN), linear discriminant analysis (LDA), and ensemble learning) to obtain an accuracy of 90.8% with SVM for classifying the citrus Huanglongbing (HLB) disease. Gu et al. [31] evaluated early detection of tomato spotted wilt virus infection in tobacco testing four ML algorithms (boosted regression tree (BRT), SVM, RF, and classification and regression tree (CART)). The BRT and RF models showed better performance (accuracies of 85.2 and 80.5%, respectively), and the CART models achieved the worst performance (72.4%). Karadağ et al. [19] used three ML algorithms to obtain accuracy rates of 100% for KNN, 97.5% for artificial neural network (ANN), and 90% for Naïve Bayes (NB) for classifying the pepper fusarium disease.

In our study, the RF, ANN, and bagged carts (BCART) models achieved the best performance (accuracies of 98.0 to 99.8%) for classifying the levels of SALB severity with the 10-fold CV 5 times method (Table 3), while the ANN and SVM models were the best (accuracy of 100.0%) with the testing dataset (Table 3 and Table 4). The BRT model had lower performance. Our results showed how the bagging ensemble method improved the CART model performance, but the boosting method had a lower impact on the regression tree performance, contrary to that report by Gu et al. [31]. The higher performance in the ANN and RF models were also demonstrated here. Nevertheless, our results showed superior performance in various tested techniques, as compared to the above studies. In addition, the potential for overfitting some models could be related to two aspects: (1) similar leaf spectral patterns associated with severity classes ‘1’ and ‘2’ (i.e., a confusing pattern or noise in the data that does not allow the learner to recognize new data) [26,28]; and (2), a lower data proportion of class ‘1’ in relation to class ‘2’ in both modeling phases (i.e., unbalanced data): 11.76% for class ‘1’ as compared to 20.58% for class ‘2’ in the test dataset, and 8.33% as compared to 15.00% in the training dataset. This probably resulted in models such as RF, BRT, and BCART being erroneously classified as class ‘2’ instead of class ‘1’ (an error rate of 25%). Despite the strength of the predictive modeling, limitations in the ML algorithms also include the requirement for a large dataset for training to achieve statistical significance [62].

Overall, our results showed a desirable ability in the tuned models to classify the SALB levels independently of phenology or genotype, similar to that reported by Sterling and Melgarejo [20], who used multiple regression techniques on spectral vegetation indices and found no influence of phenology when discriminating SALB symptoms.

### 4.4. Prediction of Photosynthetic Changes Caused by SALB

Recent studies have tested the performance of ML algorithms based on spectral reflectance for modeling photosynthesis-related traits. Fu et al. [16] tested six ML algorithms to estimate photosynthetic capacities. The least absolute shrinkage and selection operator (LASO) model achieved the highest performance (R^2^ = 0.65) with cross-validation, while SVM was the best in the testing phase (R^2^ = 0.67).

Sonobe et al. [29] tested the performance of four ML algorithms: RF, SVM, deep belief nets (DBN), and kernel-based extreme learning machine (KELM) for estimating tea leaf chlorophyll content. KELM performed best with an R^2^ of 0.93, and RMSE had 8.94 µg cm^−2^.

Boshkovski et al. [24] tested two ML algorithms to predict photosynthesis and biochemical traits in two *Phaseolus vulgaris* genotypes. The partial least squares regression (PLSR) method performed better when predicting the net photosynthetic rate (*A*) (R^2^ of 0.85, and RMSE had 2.12 µmol m^−2^ s^−1^).

Our results showed that the RF, SVM, and BRT models achieved the best performance (R^2^ ranged from 0.80 to 0.99) for predicting the four gas exchange-related traits (*A*, *E*, *g_s_*, and *WUEe*) with the 10-fold CV 5 times method (Table 5), while the RF model was the best (R^2^ ranged from 0.81 to 0.99) in the testing phase. In contrast, lower performances were evidenced in the different ML algorithms in both the training and testing phases with the five chlorophyll *a* fluorescence-related traits (*F_v_*/*F_m_*, *F_v_′*/*F_m_′*, NPQ, ETR, and *qP*) (i.e., R^2^ ranged from 0.24 to 0.52). Nevertheless, all evaluated models had significant R^2^ values (*p* < 0.05) for the nine physiological traits in the testing phase. In addition, the fitted models had higher performance than reported in other plant species, especially for predicting gas exchange-related traits, such as *A* (R^2^ of 0.99) and *WUEe* (R^2^ of 0.98), with the BRT and ANN models.

Overall, the RF and ANN models had similar and high performance for predicting SALB severity classes and leaf gas exchange-related traits such as *A* and *WUEe*, while the five ML algorithms had a lower performance for predicting photochemical traits when compared to that observed when classifying SALB levels.

## 5. Conclusions

The use of leaf hyperspectral reflectance analysis in visible and near-infrared combined with five machine learning algorithms (RF, BRT, BCART, ANN, and SVM) efficiently detected, discriminated, and classified the SALB levels, and predicted, for the first time, disease-induced photochemical and non-photochemical photosynthetic changes in young leaves on rubber tree. Our study identified 20 optimum spectral bands (9 in the green region, 3 in the yellow/orange region, 7 in the red region, and 1 in the NIR region) with the best ability for classifying SALB levels, and predicting photosynthetic alterations caused by this disease.

The RF, ANN, and BCART models achieved the best performance for classifying the SALB levels on training (accuracies of 98.0 to 99.8%) and test (accuracies of 97.1 to 100%) spectral data. In addition, the ANN and RF models had the highest performance for predicting the *A* and *WUEe* traits in the training (R^2^ ranged from 0.97 to 0.99) and testing (R^2^ ranged from 0.96 to 0.99) phases.

Our results will serve as the basis for future designs of remote detection systems for early diagnosis and monitoring of the intensity of SALB symptoms, as well as disease-induced photosynthetic limitations in rubber trees, for more effective SALB management, especially in tropical regions that have a higher incidence of this disease.

## Figures and Tables

**Table 1:** Detail of samples for each SALB severity class used for the photosynthetic and hyperspectral data analysis in *Hevea brasiliensis*.

Severity Class	Severity Range	Inoculation	Days of Sampling ^a^	Observed Cultivars	Number of Samples
0	0%	Without	0, 4, 8, 12, 16 and 20	FX 3864, FX 4098	60
		With	0	FX 3864, FX 4098	10
1	0.2–5%	With	4, 8, 12 and 16	FX 3864, FX 4098	14
2	6–15%	With	4, 8, 12, 16 and 20	FX 3864, FX 4098	25
3	18–20%	With	8, 12, 16 and 20	FX 3864	6
4	40–100%	With	12, 16 and 20	FX 3864	5
				Total	120

^a^ Days 0, 4, and 8, corresponded to B stage leaflets, and days 12, 16, and 20 to C stage leaflets.

**Table 2:** Mean values (±standard error) of nine photosynthetic traits for each SALB severity class in each leaf stage of *Hevea brasiliensis*: net CO2 assimilation rate (*A*) (µmol CO_2_ m^−2^ s^−1^), transpiration rate (*E*) (mmol H_2_O m^−2^ s^−1^), stomatal conductance to water vapor (*g_s_*) (mol H_2_O m^−2^ s^−1^), water use efficiency extrinsic (*WUEe*) (µmol CO_2_ mmol H_2_O^−1^), the maximum quantum yield of photosystem II (PSII) (*F_v_*/*F_m_*), efficiency of excitation energy capture by open PSII reaction centers (*F_v_′*/*F_m_′*), non-photochemical quenching coefficient (NPQ), electron transport rate (ETR), and photochemical quenching coefficient (*qP*).

Severity Class	Leaf Stage	*A*	*E*	*g_s_*	*WUE_e_*	*F_v_*/*F_m_*
0	B	−4.31 ± 0.33	0.63 ± 0.01	0.20 ± 0.01	−7.08 ± 0.75	0.83 ± 0.00
	C	2.99 ± 0.07	0.83 ± 0.02	0.38 ± 0.02	3.59 ± 0.07	0.83 ± 0.00
1	B	−6.76 ± 0.71	0.54 ± 0.03	0.17 ± 0.02	−12.71 ± 1.43	0.82 ± 0.00
	C	2.40 ± 0.25	0.63 ± 0.07	0.20 ± 0.08	3.72 ± 0.24	0.82 ± 0.02
2	B	−6.99 ± 0.89	0.54 ± 0.03	0.19 ± 0.03	−13.98 ± 1.79	0.81 ± 0.01
	C	2.75 ± 0.10	0.71 ± 0.02	0.18 ± 0.03	3.89 ± 0.10	0.81 ± 0.01
3	B	−11.90 ± 1.66	0.42 ± 0.06	0.15 ± 0.06	−28.06 ± 3.36	0.75 ± 0.01
	C	1.45 ± 0.21	0.44 ± 0.06	0.16 ± 0.07	3.23 ± 0.21	0.79 ± 0.02
4	B	-	-	-	-	-
	C	1.01 ± 0.19	0.37 ± 0.05	0.14 ± 0.06	2.51 ± 0.19	0.72 ± 0.02
**Severity Class**	**Leaf Stage**	***F_v_′*/*F_m_′***	**NPQ**	**ETR**	***qP***	
0	B	0.76 ± 0.00	0.01 ± 0.00	0.19 ± 0.01	0.06 ± 0.00	
	C	0.74 ± 0.00	0.01 ± 0.00	0.29 ± 0.01	0.11 ± 0.01	
1	B	0.75 ± 0.01	0.02 ± 0.00	0.16 ± 0.03	0.05 ± 0.01	
	C	0.75 ± 0.02	0.02 ± 0.00	0.28 ± 0.06	0.10 ± 0.03	
2	B	0.73 ± 0.01	0.02 ± 0.00	0.15 ± 0.04	0.05 ± 0.01	
	C	0.71 ± 0.00	0.03 ± 0.00	0.29 ± 0.02	0.12 ± 0.01	
3	B	0.52 ± 0.03	0.03 ± 0.00	0.03 ± 0.07	0.01 ± 0.02	
	C	0.71 ± 0.01	0.03 ± 0.00	0.16 ± 0.05	0.06 ± 0.02	
4	B	-	-	-	-	
	C	0.70 ± 0.01	0.04 ± 0.00	0.19 ± 0.04	0.07 ± 0.02	

- Does not apply (severity class not reported in the B stage leaflets).

**Table 3:** Results of five models used to classify different SALB severity classes in the training and testing phases.

Model	Training		Testing	
	Accuracy (%)	Kappa Coefficient	Accuracy (%)	Kappa Coefficient
RF	99.8	0.99	97.1	0.95
BRT	95.6	0.93	94.1	0.89
BCART	98.0	0.97	97.1	0.95
ANN	98.1	0.97	100.0	1.00
SVM	96.7	0.95	100.0	1.00

RF, random forest; BRT, boosted regression tree; BCART, bagged carts; ANN, artificial neural Network; SVM, support vector machine.

**Table 4:** Statistics by class of five models are used to classify different SALB severity classes in the testing phase.

Model	Severity Class	Sensitivity	Specificity	Positive Predictive Value	Negative Predictive Value	Balance Accuracy (%)
RF	0	1.00	1.00	1.00	1.00	100.0
	1	0.75	1.00	1.00	0.97	87.5
	2	1.00	0.96	0.88	1.00	98.1
	3	1.00	1.00	1.00	1.00	100.0
	4	1.00	1.00	1.00	1.00	100.0
BRT	0	1.00	1.00	1.00	1.00	100.0
	1	0.75	1.00	1.00	0.97	87.5
	2	1.00	0.96	0.88	1.00	98.1
	3	1.00	1.00	1.00	1.00	100.0
	4	1.00	0.97	0.50	1.00	98.4
BCART	0	1.00	1.00	1.00	1.00	100.0
	1	0.75	1.00	1.00	0.97	87.5
	2	1.00	0.96	0.88	1.00	98.1
	3	1.00	1.00	1.00	1.00	100.0
	4	1.00	1.00	1.00	1.00	100.0
ANN	0	1.00	1.00	1.00	1.00	100.0
	1	1.00	1.00	1.00	1.00	100.0
	2	1.00	1.00	1.00	1.00	100.0
	3	1.00	1.00	1.00	1.00	100.0
	4	1.00	1.00	1.00	1.00	100.0
SVM	0	1.00	1.00	1.00	1.00	100.0
	1	1.00	1.00	1.00	1.00	100.0
	2	1.00	1.00	1.00	1.00	100.0
	3	1.00	1.00	1.00	1.00	100.0
	4	1.00	1.00	1.00	1.00	100.0

RF, random forest; BRT, boosted regression tree; BCART, bagged carts; ANN, artificial neural Network; SVM, support vector machine.

**Table 5:** Results of five models used to predict nine photosynthetic traits of *Hevea brasiliensis*: net CO_2_ assimilation rate (*A*) (µmol CO_2_ m^−2^ s^−1^), transpiration rate (*E*) (mmol H_2_O m^−2^ s^−1^), stomatal conductance to water vapor (gs) (mol H_2_O m^−2^ s^−1^), water use efficiency extrinsic (*WUE_e_*) (µmol CO_2_ mmol H_2_O^−1^), the maximum quantum yield of photosystem II (PSII) (*F_v_*/*F_m_*), efficiency of excitation energy capture by open PSII reaction centers (*F_v_′*/*F_m_′*), non-photochemical quenching coefficient (NPQ), electron transport rate (ETR), and photochemical quenching coefficient (*qP*).

Trait	Model	Training		Testing		Trait	Model	Training		Testing	
		RMSE	R^2^	RMSE	R^2^			RMSE	R^2^	RMSE	R^2^
*A*	RF	0.672	0.98	0.407	0.99	*F_v_′*/*F_m_′*	RF	0.042	0.52	0.047	0.14
	BRT	0.539	0.99	0.716	0.98		BRT	0.046	0.43	0.045	0.19
	BCART	1.505	0.90	0.956	0.96		BCART	0.046	0.41	0.045	0.15
	ANN	0.422	0.99	0.566	0.99		ANN	0.050	0.38	0.039	0.28
	SVM	0.627	0.98	0.893	0.97		SVM	0.045	0.42	0.042	0.20
*E*	RF	0.076	0.78	0.083	0.82	NPQ	RF	0.010	0.35	0.011	0.29
	BRT	0.076	0.78	0.086	0.78		BRT	0.010	0.37	0.013	0.24
	BCART	0.089	0.72	0.104	0.72		BCART	0.011	0.24	0.012	0.18
	ANN	0.082	0.73	0.097	0.76		ANN	0.010	0.29	0.015	0.16
	SVM	0.071	0.80	0.067	0.89		SVM	0.010	0.28	0.013	0.20
*g_s_*	RF	0.061	0.85	0.045	0.81	ETR	RF	0.095	0.48	0.091	0.39
	BRT	0.064	0.82	0.053	0.75		BRT	0.095	0.47	0.116	0.40
	BCART	0.070	0.80	0.046	0.80		BCART	0.105	0.34	0.100	0.25
	ANN	0.062	0.86	0.049	0.80		ANN	0.106	0.30	0.099	0.27
	SVM	0.057	0.88	0.046	0.79		SVM	0.098	0.43	0.113	0.42
*WUE_e_*	RF	1.571	0.97	1.620	0.97	*qP*	RF	0.037	0.46	0.042	0.74
	BRT	1.214	0.98	2.500	0.92		BRT	0.037	0.39	0.054	0.60
	BCART	3.179	0.87	2.367	0.92		BCART	0.037	0.39	0.058	0.38
	ANN	1.274	0.98	1.839	0.96		ANN	0.038	0.33	0.061	0.32
	SVM	1.467	0.97	2.091	0.90		SVM	0.039	0.37	0.042	0.73
*F_v_*/*F_m_*	RF	0.034	0.43	0.033	0.38						
	BRT	0.036	0.36	0.033	0.33						
	BCART	0.037	0.33	0.032	0.37						
	ANN	0.035	0.42	0.042	0.30						
	SVM	0.035	0.34	0.033	0.43						

RF, random forest; BRT, boosted regression tree; BCART, bagged carts; ANN, artificial neural network; SVM, support vector machine; RMSE, root-mean-square error; R^2^, determination coefficient.

## Data Availability

Data are available from the authors upon request.

## Supplementary Materials

The following are available online at https://www.mdpi.com/article/10.3390/plants11030329/s1, Table S1: Models features and parameter tuning for five machine learning algorithms; Figure S1: Dot plots for the accuracy (classification) and RMSE (numerical prediction) metrics using the 10-fold cross-validation repeated five times method for five machine learning models. Each line represents the mean and 95% confidence interval. (a) Severity class, (b) net CO_2_ assimilation rate (*A*) (µmol CO_2_ m^−2^ s^−1^), (c) transpiration rate (*E*) (mmol H_2_O m^−2^ s^−1^), (d) stomatal conductance to water vapor (*g_s_*) (mol H_2_O m^−2^ s^−1^), (e) water use efficiency extrinsic (*WUE_e_*), (f) maximum quantum yield of photosystem II (PSII) (*F_v_*/*F_m_*), (g) efficiency of excitation energy capture by open PSII reaction centers (*F_v_′*/*F_m_′*), (h) non-photochemical quenching coefficient (NPQ), (i) electron transport rate (ETR), and (j) photochemical quenching coefficient (*qP*); Figure S2: Predicted vs. measured values plots of the testing datasets results for five machine learning models used to predict nine photosynthetic traits: net CO_2_ assimilation rate (*A*) (µmol CO_2_ m^−2^ s^−1^), (c) transpiration rate (*E*) (mmol H_2_O m^−2^ s^−1^), (d) stomatal conductance to water vapor (*g_s_*) (mol H_2_O m^−2^ s^−1^), (e) water use efficiency extrinsic (*WUE_e_*), (f) maximum quantum yield of photosystem II (PSII) (*F_v_*/*F_m_*), (g) efficiency of excitation energy capture by open PSII reaction centers (*F_v_′*/*F_m_′*), (h) non-photochemical quenching coefficient (NPQ), (i) electron transport rate (ETR), and (j) photochemical quenching coefficient (*qP*); (a) random forest (RF), (b) boosted regression tree (BRT), (c) bagged classification and regression trees (BCART), (d) artificial neural network (ANN), and (e) support vector machine (SVM).

## References

[1] Venkatachalam P., Geetha N., Sangeetha P., Thulaseedharan A. (2013). Natural rubber producing plants: An overview. Afr. J. Biotechnol..

[2] IRSG, International Rubber Study Group (2019). Rubber Statiscal Bulletin.

[3] Gonçalves P.D.S., Ortolani A.A., Cardoso M. (1997). Melhoramento Genetico da Seringueira: Uma Revisão.

[4] Hora Júnior B.T., De Macedo D.M., Barreto R.W., Evans H.C., Raimundo C., Mattos R., Maffia L.A., Mizubuti E.S.G. (2014). Erasing the Past: A New Identity for the Damoclean Pathogen Causing South American Leaf Blight of Rubber. PLoS ONE.

[5] Sterling A., Melgarejo L.M. (2018). Leaf gas exchange and chlorophyll a fluorescence in *Hevea brasiliensis* in response to *Pseudocercospora ulei* infection. Physiol. Mol. Plant. Pathol..

[6] Lieberei R. (2007). South American Leaf Blight of the Rubber Tree (*Hevea* spp.): New Steps in Plant Domestication using Physiological Features and Molecular Markers. Ann. Bot..

[7] Gasparotto L., Ferreira F.A., Santos Á.F., Rezende-Pereira J.C., Furtado E.L., Gasparotto L., Rezende-Pereira J.C. (2012). Capítulo 3. Doenças das folhas. Doenças da Seringueira no Brasil.

[8] Guyot J., Le Guen V. (2018). A Review of a Century of Studies on South American Leaf Blight of the Rubber Tree. Plant Dis..

[9] Khaled A.Y., Aziz S.A., Bejo S.K., Nawi N.M., Seman I.A., Onwude D.I. (2018). Early detection of diseases in plant tissue using spectroscopy—Applications and limitations. Appl. Spectrosc. Rev..

[10] Mahlein A.-K., Rumpf T., Welke P., Dehne H.-W., Plümer L., Steiner U., Oerke E.-C. (2013). Development of spectral indices for detecting and identifying plant diseases. Remote Sens. Environ..

[11] Zhang J., Huang Y., Pu R., Gonzalez-Moreno P., Yuan L., Wu K., Huang W. (2019). Monitoring plant diseases and pests through remote sensing technology: A review. Comput. Electron. Agric..

[12] Mahlein A.-K. (2016). Plant Disease Detection by Imaging Sensors—Parallels and Specific Demands for Precision Agriculture and Plant Phenotyping. Plant Dis..

[13] Feng L., Chen S., Zhang C., Zhang Y., He Y. (2021). A comprehensive review on recent applications of unmanned aerial vehicle remote sensing with various sensors for high-throughput plant phenotyping. Comput. Electron. Agric..

[14] Behmann J., Mahlein A.K., Rumpf T., Römer C., Plümer L. (2015). A review of advanced machine learning methods for the detection of biotic stress in precision crop protection. Precis. Agric..

[15] Marín-Ortiz J.C., Gutierrez-Toro N., Botero-Fernández V., Hoyos-Carvajal L.M. (2020). Linking physiological parameters with visible/near-infrared leaf reflectance in the incubation period of vascular wilt disease. Saudi J. Biol. Sci..

[16] Fu P., Meacham-Hensold K., Guan K., Bernacchi C.J. (2019). Hyperspectral leaf reflectance as proxy for photosynthetic capacities: An ensemble approach based on multiple machine learning algorithms. Front. Plant Sci..

[17] Sun P., Grignetti A., Liu S., Casacchia R., Salvatori R., Pietrini F., Loreto F., Centritto M. (2008). Associated changes in physiological parameters and spectral reflectance indices in olive (*Olea europaea* L.) leaves in response to different levels of water stress. Int. J. Remote Sens..

[18] Heim R.H.J., Wright I.J., Chang H.C., Carnegie A.J., Pegg G.S., Lancaster E.K., Falster D.S., Oldeland J. (2018). Detecting myrtle rust (*Austropuccinia psidii*) on lemon myrtle trees using spectral signatures and machine learning. Plant. Pathol..

[19] Karadağ K., Tenekeci M.E., Taşaltın R., Bilgili A. (2020). Detection of pepper fusarium disease using machine learning algorithms based on spectral reflectance. Sustain. Comput. Inform. Syst..

[20] Sterling A., Melgarejo L.M. (2020). Leaf spectral reflectance of *Hevea brasiliensis* in response to *Pseudocercospora ulei*. Eur. J. Plant Pathol..

[21] Furlanetto R.H., Nanni M.R., Mizuno M.S., Crusiol L.G.T., da Silva C.R. (2021). Identification and classification of Asian soybean rust using leaf-based hyperspectral reflectance. Int. J. Remote Sens..

[22] Deng X., Huang Z., Zheng Z., Lan Y., Dai F. (2019). Field detection and classification of citrus Huanglongbing based on hyperspectral reflectance. Comput. Electron. Agric..

[23] El-Hendawy S., Al-Suhaibani N., Hassan W., Tahir M., Schmidhalter U. (2017). Hyperspectral reflectance sensing to assess the growth and photosynthetic properties of wheat cultivars exposed to different irrigation rates in an irrigated arid region. PLoS ONE.

[24] Boshkovski B., Tzerakis C., Doupis G., Zapolska A., Kalaitzidis C., Koubouris G. (2021). Relationship between physiological and biochemical measurements with spectral reflectance for two *Phaseolus vulgaris* L. genotypes under multiple stress. Int. J. Remote Sens..

[25] Fang S., Cui R., Wang Y., Zhao Y., Yu K., Jiang A. (2021). Application of multiple spectral systems for the tree disease detection: A review. Appl. Spectrosc. Rev..

[26] Kuhn M., Johnson K. (2013). Applied Predictive Modeling.

[27] Golhani K., Balasundram S.K., Vadamalai G., Pradhan B. (2018). A review of neural networks in plant disease detection using hyperspectral data. Inf. Process. Agric..

[28] Lantz B. (2019). Machine Learning with R: Expert Techniques for Predictive Modeling.

[29] Sonobe R., Hirono Y., Oi A. (2020). Non-destructive detection of tea leaf chlorophyll content using hyperspectral reflectance and machine learning algorithms. Plants.

[30] Pane C., Manganiello G., Nicastro N., Cardi T., Carotenuto F. (2021). Powdery Mildew Caused by *Erysiphe cruciferarum* on Wild Rocket (*Diplotaxis tenuifolia*): Hyperspectral Imaging and Machine Learning Modeling for Non-Destructive Disease Detection. Agriculture.

[31] Gu Q., Sheng L., Zhang T., Lu Y., Zhang Z., Zheng K., Hu H., Zhou H. (2019). Early detection of tomato spotted wilt virus infection in tobacco using the hyperspectral imaging technique and machine learning algorithms. Comput. Electron. Agric..

[32] Breiman L. (2001). Random Forests. Mach. Learn..

[33] Domingos da Silva A.L., Alves Filho E.G., Silva L.M.A., Tavares O.C.H., Pereira M.G., de Campos T., da Silva L.M. (2021). Near infrared spectroscopy to rapid assess the rubber tree clone and the influence of maturation and disease at the leaves. Microchem. J..

[34] Mattos C.R.R., Garcia D., Pinard F., Le Guen V. (2003). Variabilidade de isolados de *Microcyclus ulei* no Sudeste da Bahia. Fitopatol. Bras..

[35] Sterling A., Melgarejo L.M. (2021). Photosynthetic performance of *Hevea brasiliensis* affected by South American Leaf Blight under field conditions. Eur. J. Plant Pathol..

[36] Rivano F., Martinez M., Cevallos V., Cilas C. (2010). Assessing resistance of rubber tree clones to *Microcyclus ulei* in large-scale clone trials in Ecuador: A less time-consuming field method. Eur. J. Plant Pathol..

[37] IGAC, Instituto Geográfico Agustín Codazzi (2014). Estudio General de Suelos y Zonificación de Tierras Departamento de Caquetá.

[38] Feldmann F., Junqueira N.T., Meier U. (2005). Phenological Growth Stages of the Rubber Tree Hevea Brasiliensis (Willd. ex Adr. de Juss.) Muell.-Arg.: Codification and Description According to the BBCH Scale.

[39] Torres C. (1999). Manual Para el Cultivo del Caucho en la Amazonia.

[40] Sterling A., Salas-Tobón Y., Virgüez-Díaz Y., Vargas-Losada M., Obando-Guzmán J., Sterling A., Rodríguez C. (2014). Evaluación fitosanitaria con énfasis en la reacción a Microcyclus ulei de tres clones de caucho (*Hevea brasiliensis*) en sistema agroforestal con copoazú (*Theobroma grandiflorum*) y plátano hartón (Musa AAB). Agroforestería en el Caquetá: Clones Promisorios de Caucho en Asocio con Copoazú y Plátano Hartón con Potencial para la Amazonia Colombiana.

[41] Sterling A., Melgarejo L.M. (2014). Variación temporal a *Microcyclus ulei* en los clones de caucho FX 3864 y FX 4098 en condiciones controladas. Rev. Colomb. Biotecnol..

[42] Bajwa S.G., Rupe J.C., Mason J. (2017). Soybean disease monitoring with leaf reflectance. Remote Sens..

[43] Rossel R.A.V. (2008). ParLeS: Software for chemometric analysis of spectroscopic data. Chemom. Intell. Lab. Syst..

[44] Riefolo C., Antelmi I., Castrignanò A., Ruggieri S., Galeone C., Belmonte A., Muolo M.R., Ranieri N.A., Labarile R., Gadaleta G. (2021). Assessment of the hyperspectral data analysis as a tool to diagnose *Xylella fastidiosa* in the asymptomatic leaves of olive plants. Plants.

[45] Zhao J., Fang Y., Chu G., Yan H., Hu L., Huang L. (2020). Identification of Leaf-Scale Wheat Powdery Mildew (*Blumeria graminis* f. sp. Tritici) Combining Hyperspectral Imaging and an SVM Classifier. Plants.

[46] Di Rienzo J.A., Casanoves F., Balzarini M.G., Gonzalez L., Tablada M., Robledo C.W. (2020). InfoStat.

[47] Kandpal K.C., Kumar S., Venkat G.S., Meena R., Pal P.K., Kumar A. (2021). Onsite age discrimination of an endangered medicinal and aromatic plant species Valeriana jatamansi using field hyperspectral remote sensing and machine learning techniques. Int. J. Remote Sens..

[48] Hennessy A., Clarke K., Lewis M. (2020). Hyperspectral Classification of Plants: A Review of Waveband Selection Generalisability. Remote Sens..

[49] Holden H., LeDrew E. (1998). Spectral discrimination of healthy and non-healthy corals based on cluster analysis, principal components analysis, and derivative spectroscopy. Remote Sens. Environ..

[50] Bruce P., Bruce A. (2017). Practical Statistics for Data Scientists.

[51] Wang F., Huang J., Zhou Q., Wang X. (2009). Optimal waveband identification for estimation of leaf area index of paddy rice. J. Zhejiang Univ. Sci. B.

[52] James G., Witten D., Hastie T., Tibshirani R. (2013). An Introduction to Statistical Learning.

[53] (2020). R Core Team R: A Language and Environment for Statistical Computing.

[54] (2020). RStudio.

[55] Kuhn M., Wing J., Weston S., Williams A., Keefer C., Engelhardt A., Cooper T., Mayer Z., Kenkel B., Team R.C. (2020). Package ‘Caret’: Classification and Regression Training Version 6.0-86.

[56] Ramasubramanian K., Singh A. (2017). Machine Learning Using R—A Comprehensive Guide to Machine Learning.

[57] Maxwell A.E., Warner T.A., Fang F. (2018). Implementation of machine-learning classification in remote sensing: An applied review. Int. J. Remote Sens..

[58] Elith J., Leathwick J.R., Hastie T. (2008). A working guide to boosted regression trees. J. Anim. Ecol..

[59] Breiman L. (1996). Bagging predictors. Mach. Learn..

[60] Ghimire B., Rogan J., Galiano V.R., Panday P., Neeti N. (2012). An Evaluation of Bagging, Boosting, and Random Forests for Land-Cover Classification in Cape Cod, Massachusetts, USA. GIScience Remote Sens..

[61] Hiddar H., Rehman S., Lakew B., Verma R.P.S., Al-Jaboobi M., Moulakat A., Kehel Z., Filali-Maltouf A., Baum M., Amri A. (2021). Assessment and modeling using machine learning of resistance to scald (*Rhynchosporium commune*) in two specific barley genetic resources subsets. Sci. Rep..

[62] Houshmandfar A., O’Leary G., Fitzgerald G.J., Chen Y., Tausz-Posch S., Benke K., Uddin S., Tausz M. (2021). Machine learning produces higher prediction accuracy than the Jarvis-type model of climatic control on stomatal conductance in a dryland wheat agro-ecosystem. Agric. For. Meteorol..

[63] Hothorn T., Leisch F., Zeileis A., Hornik K. (2005). The Design and Analysis of Benchmark Experiments. J. Comput. Graph. Stat..

[64] Jing L., Jinbao J., Yunhao C., Yuanyuan W., Wei S., Wenjiang H. (2007). Using hyperspectral indices to estimate foliar chlorophyll a concentrations of winter wheat under yellow rust stress. N. Z. J. Agric. Res..

[65] Sterling A., Rodríguez C.H. (2018). Estrategias de Manejo para las Principales Enfermedades y Plagas del Cultivo del Caucho con Énfasis en la Amazonia Colombiana.

[66] Pietrzykowski E., Stone C., Pinkard E., Mohammed C. (2006). Effects of Mycosphaerella leaf disease on the spectral reflectance properties of juvenile *Eucalyptus globulus* foliage. For. Pathol..

