# Supplementary material for: Prediction of South American Leaf Blight and Disease-Induced Photosynthetic Changes in Rubber Tree, Using Machine Learning Techniques on Leaf Hyperspectral Reflectance
Source: Plants (Basel). 2022 Jan 26;11(3):329. doi: 10.3390/plants11030329 (PMC8840432; doi:10.3390/plants11030329)
Supplement: Supplementary file 1 [file plants-11-00329-s001.zip › plants-1505213-supplementary.pdf]

- **Supplementary Materials**

Prediction of South American Leaf Blight and Disease-Induced Photosynthetic Changes in Rubber Tree, Using Machine Learning Techniques on Leaf Hyperspectral Reflectance

Armando Sterling [1,2]*, Julio A. Di Rienzo [2]

[1] Phytopathology Laboratory, Instituto Amazónico de Investigaciones Científicas SINCHI – Mycology Research Group GINMUA, Programa de Biología, Facultad de Ciencias Básicas, Universidad de la Amazonía, Florencia, 180001, Colombia

[2] InfoStat Transfer Center, Facultad de Ciencias Agropecuarias, Universidad Nacional de Córdoba, 5016, Córdoba, Argentina; dirienzo@agro.unc.edu.ar (J.A.D.R.)

* Correspondence: asterling@sinchi.org.co (A.S.); Tel.: +57-310-786-2496

Supplementary Tables

Table S1. Models features and parameter tuning for five machine learning algorithms

Algorithm	Train model[a]	Tuning parameters	Predicted variable	Model' final hyperparameters
RF	rf	(i) 10-fold CV 5 times; (ii) number of randomly selected predictors (mtry) = seq(2, 20, by =2); (iii) number of trees =1000; (iv) tuneLength = 10; (v) importance = TRUE	Severity class	mtry = 8
			A	mtry = 20
			E	mtry = 20
			gs	mtry = 10
			WUEe	mtry = 10
			Fv/*Fm*	mtry = 4
			Fv'/*Fm'*	mtry = 2
			NPQ	mtry = 18
			ETR	mtry = 20
			qP	mtry = 10
BRT	gbm	(i) 10-fold CV 5 times; (ii) max tree depth (1, 3, 5 and 7); (iii) number of trees (100 to 1000, by 50); (iv) minimal terminal node size (10); shrinkage (0.01 and 0.1)	Severity class	number of trees = 100; shrinkage = 0.1; max tree depth = 1
			A	number of trees = 1000; shrinkage = 0.1; max tree depth = 5
			E	number of trees = 850; shrinkage = 0.1; max tree depth = 1
			gs	number of trees = 450; shrinkage = 0.1; max tree depth = 7
			WUEe	number of trees = 950; shrinkage = 0.1; max tree depth = 7
			Fv/*Fm*	number of trees = 1000; shrinkage = 0.01; max tree depth = 1
			Fv'/*Fm'*	number of trees = 300; shrinkage = 0.1; max tree depth = 5
			NPQ	number of trees = 1000; shrinkage = 0.1; max tree depth = 1

			ETR	number of trees = 950; shrinkage = 0.1; max tree depth = 7
			qP	number of trees = 700; shrinkage = 0.01; max tree depth = 7
BCART	treebag	(i) 10-fold CV 5 times; (ii) No other tuning parameters for this model	Severity class	-
			A	-
			E	-
			gs	-
			WUEe	-
			Fv/*Fm*	-
			Fv'/*Fm'*	-
			NPQ	-
			ETR	-
			qP	-
ANN	nnet	(i) 10-fold CV 5 times; (ii) size (1 to 9, by 2); (iii) decay (0.001, 0.01 and 0.1); (iv) preprocessing (center and scale); (v) maxit (1000); (vi) linout = TRUE; (vii) trace = FALSE	Severity class	size = 5; decay = 0.001
			A	size = 3; decay = 0.01
			E	size = 9; decay = 0.1
			gs	size = 7; decay = 0.01
			WUEe	size = 3; decay = 0.1
			Fv/*Fm*	size = 7; decay = 0.001
			Fv'/*Fm'*	size = 7; decay = 0.01
			NPQ	size = 3; decay = 0.001
			ETR	size = 1; decay = 0.1
			qP	size = 3; decay = 0.1
SVM	svmRadial	(i) 10-fold CV 5 times; (ii) sigma by default (i.e., sigma was held constant at a value of 0.1792956); (iii) cost (C) (2 to 500, by 2); (iv) tuneLength (15); (v) preprocessing (center and scale)	Severity class	C = 8
			A	C = 32
			E	C = 2
			gs	C = 4
			WUEe	C = 32
			Fv/*Fm*	C = 2
			Fv'/*Fm'*	C = 2
			NPQ	C = 4
			ETR	C = 256
			qP	C = 4

[a]Train model from R Package caret

- No appply

Random Forest (RF), Boosted Regression Tree (BRT), Bagged Classification and Regression Tree (BCART), Artificial Neural Network (ANN), Support Vector Machine (SVM), Net CO_2 assimilation rate (*A*) (μmol CO_2 m^{-2} s^{-1}), transpiration rate (*E*) (mmol H_2O m^{-2} s^{-1}), stomatal conductance to water vapor (g_s) (mol H_2O m^{-2} s^{-1}), water use efficiency extrinsic (WUE_e) (μmol CO_2 mmol H_2O^{-1}), maximum quantum yield of photosystem II (PSII) (F_v/F_m), efficiency of excitation energy capture by open PSII reaction centers (F_v'/F_m'), non-photochemical quenching coefficient (NPQ), electron transport rate (ETR), and photochemical quenching coefficient (*qP*).

Supplementary Figures

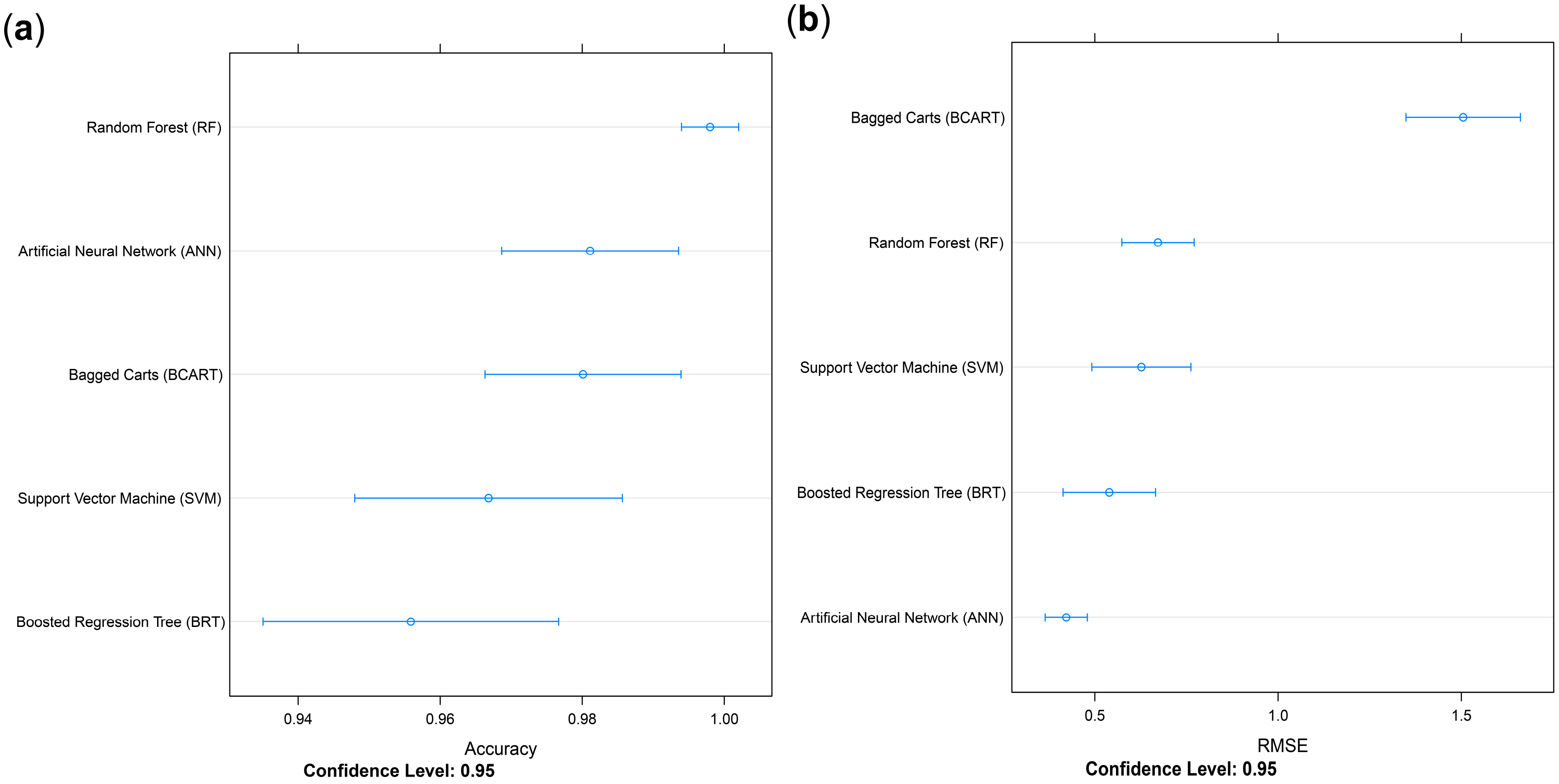

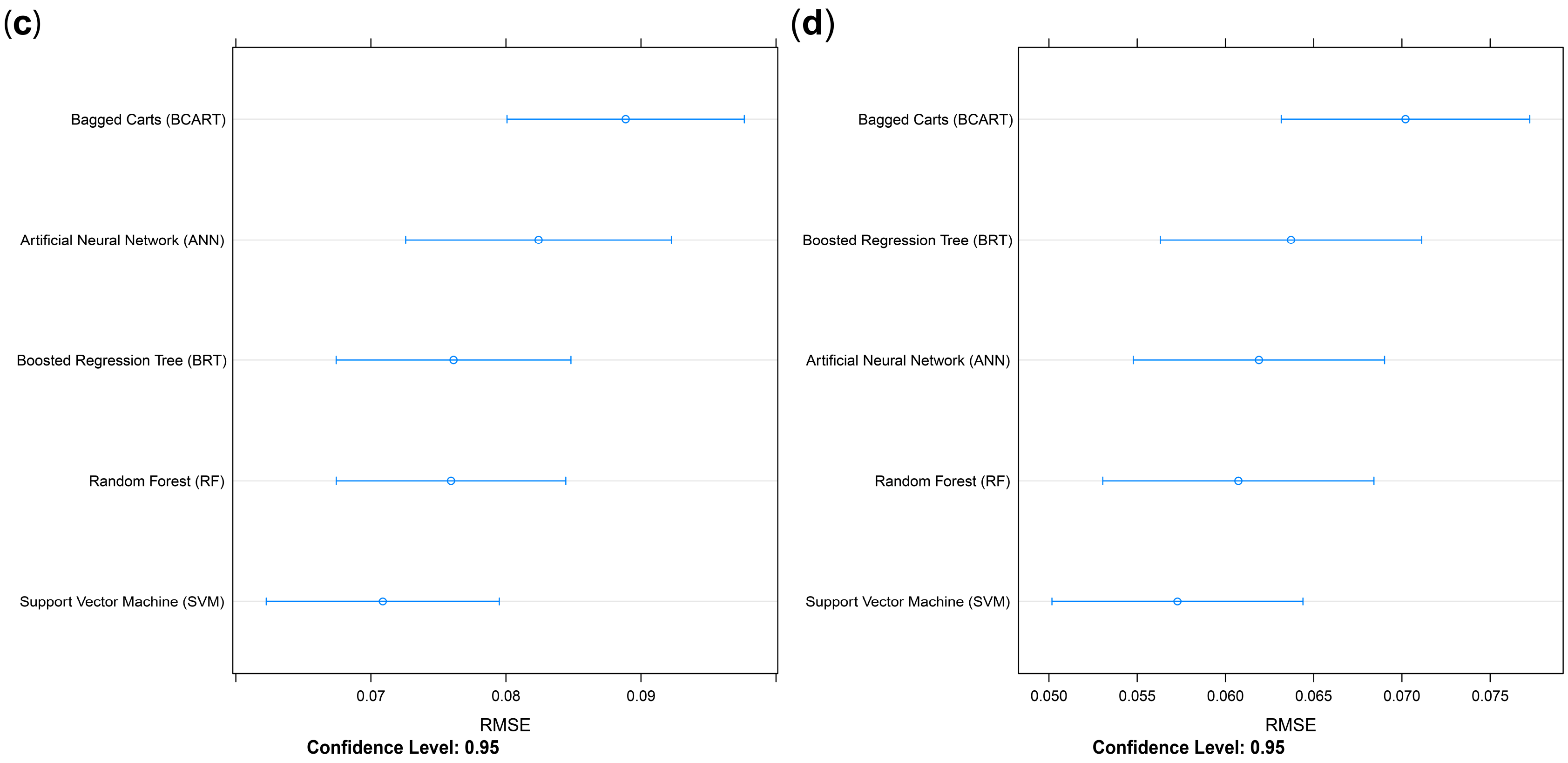

(c)
Bagged Carts (BCART)
Artificial Neural Network (ANN)
Boosted Regression Tree (BRT)
Random Forest (RF)
Support Vector Machine (SVM)
0.07
0.08
0.09
RMSE
Confidence Level: 0.95
(d)
Bagged Carts (BCART)
Boosted Regression Tree (BRT)
Artificial Neural Network (ANN)
Random Forest (RF)
Support Vector Machine (SVM)
0.050
0.055
0.060
0.065
0.070
0.075
RMSE
Confidence Level: 0.95

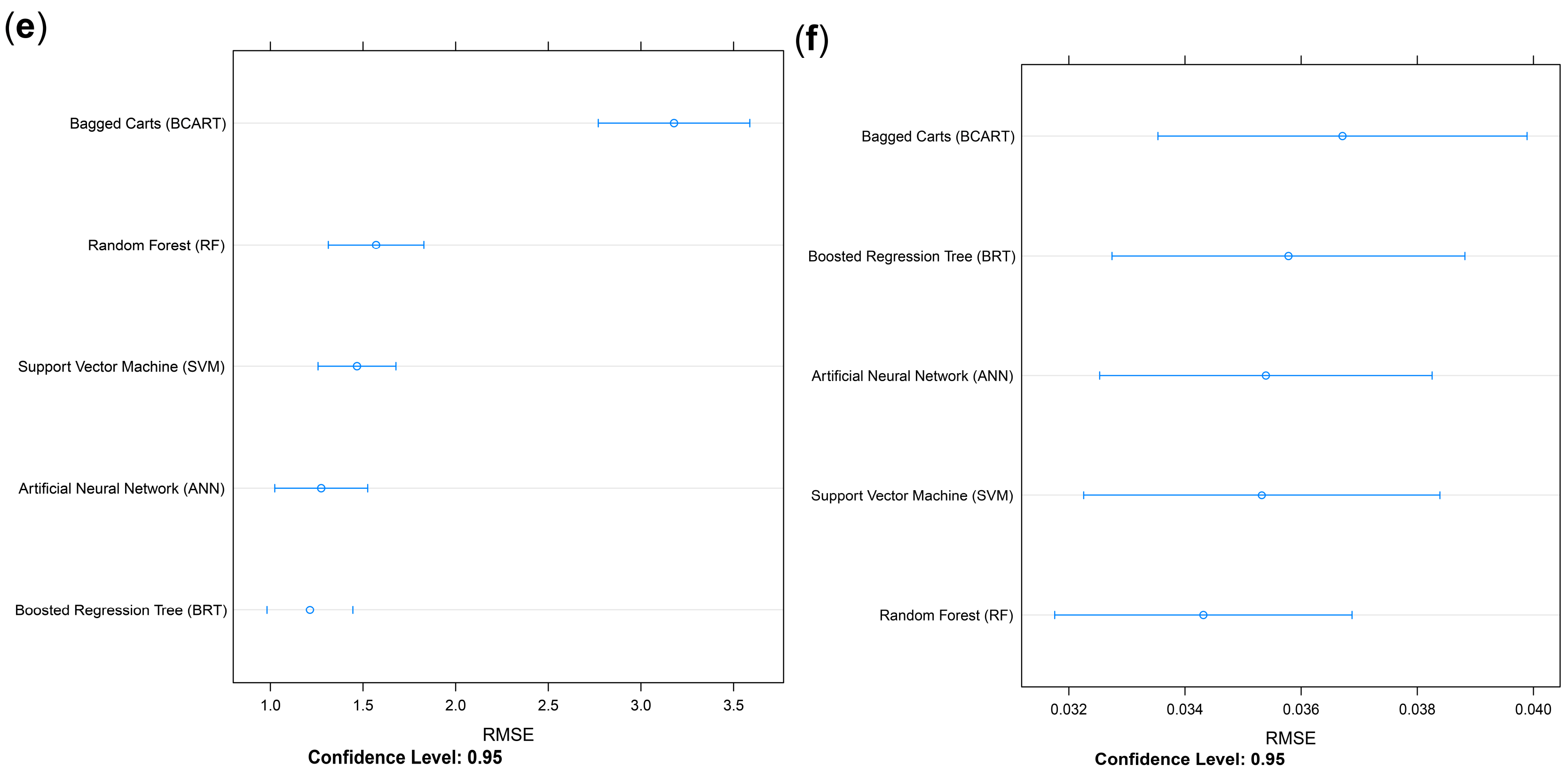
(e)
Bagged Carts (BCART)
Random Forest (RF)
Support Vector Machine (SVM)
Artificial Neural Network (ANN)
Boosted Regression Tree (BRT)
1.0
1.5
2.0
2.5
3.0
3.5
RMSE
Confidence Level: 0.95
(f)
Bagged Carts (BCART)
Boosted Regression Tree (BRT)
Artificial Neural Network (ANN)
Support Vector Machine (SVM)
Random Forest (RF)
0.032
0.034
0.036
0.038
0.040
RMSE
Confidence Level: 0.95

(g)

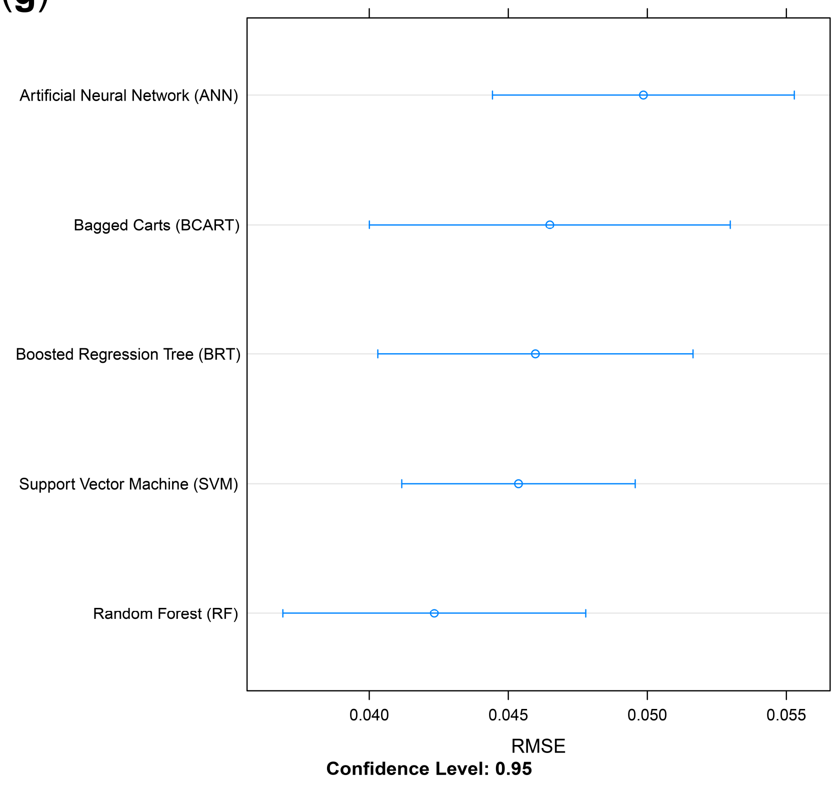

Artificial Neural Network (ANN)
Bagged Carts (BCART)
Boosted Regression Tree (BRT)
Support Vector Machine (SVM)
Random Forest (RF)
0.040
0.045
0.050
0.055
RMSE
Confidence Level: 0.95

(h)

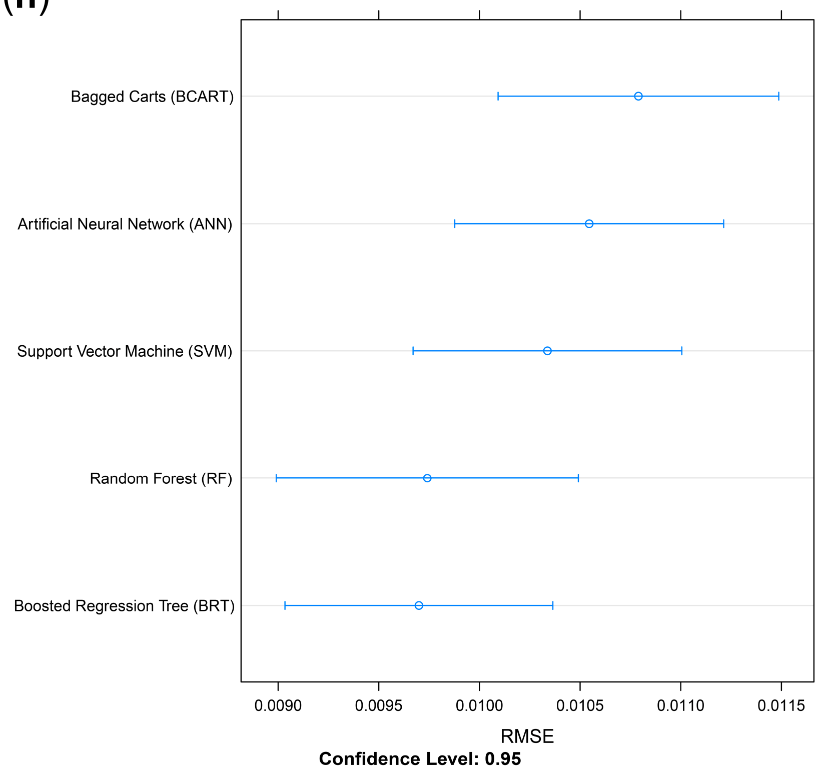

Bagged Carts (BCART)
Artificial Neural Network (ANN)
Support Vector Machine (SVM)
Random Forest (RF)
Boosted Regression Tree (BRT)
0.0090
0.0095
0.0100
0.0105
0.0110
0.0115
RMSE
Confidence Level: 0.95

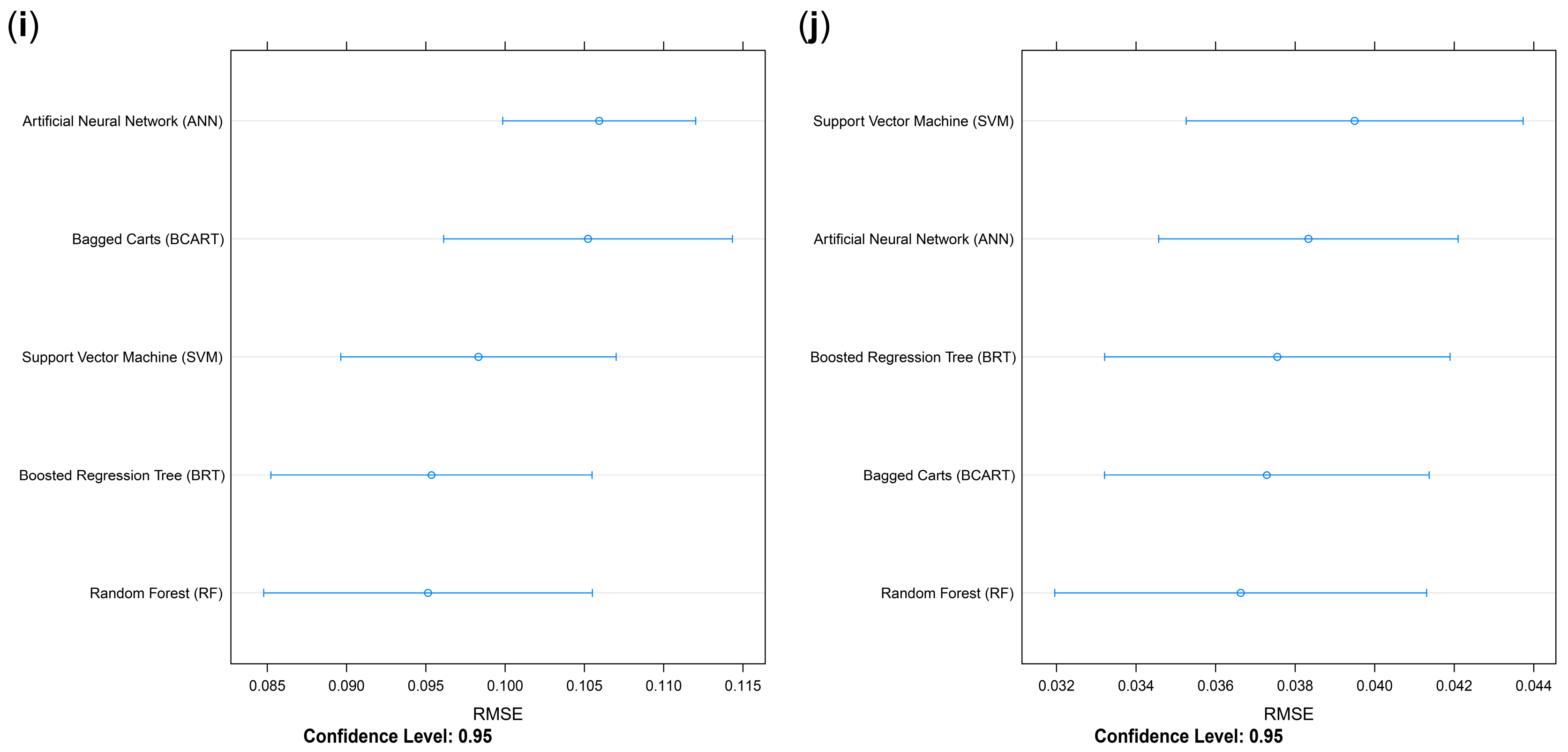

Figure S1. Dot plots for the Accuracy (classification) and RMSE (numerical prediction) metrics using the 10-fold cross-validation repeated 5 times method for five machine learning models. Each line represents the mean and 95% confidence interval. (**a**) Severity class, (**b**) net CO_2 assimilation rate (A) (μmol CO_2 m^{-2} s^{-1}), (**c**) transpiration rate (E) (mmol H_2O m^{-2} s^{-1}), (**d**) stomatal conductance to water vapor (g_s) (mol H_2O m^{-2} s^{-1}), (**e**) water use efficiency extrinsic (WUE_e), (**f**) maximum quantum yield of photosystem II (PSII) (F_v/F_m), (**g**) efficiency of excitation energy capture by open PSII reaction centers (F_v'/F_m'), (**h**) non-photochemical quenching coefficient (NPQ), (**i**) electron transport rate (ETR), and (**j**) photochemical quenching coefficient (qP)

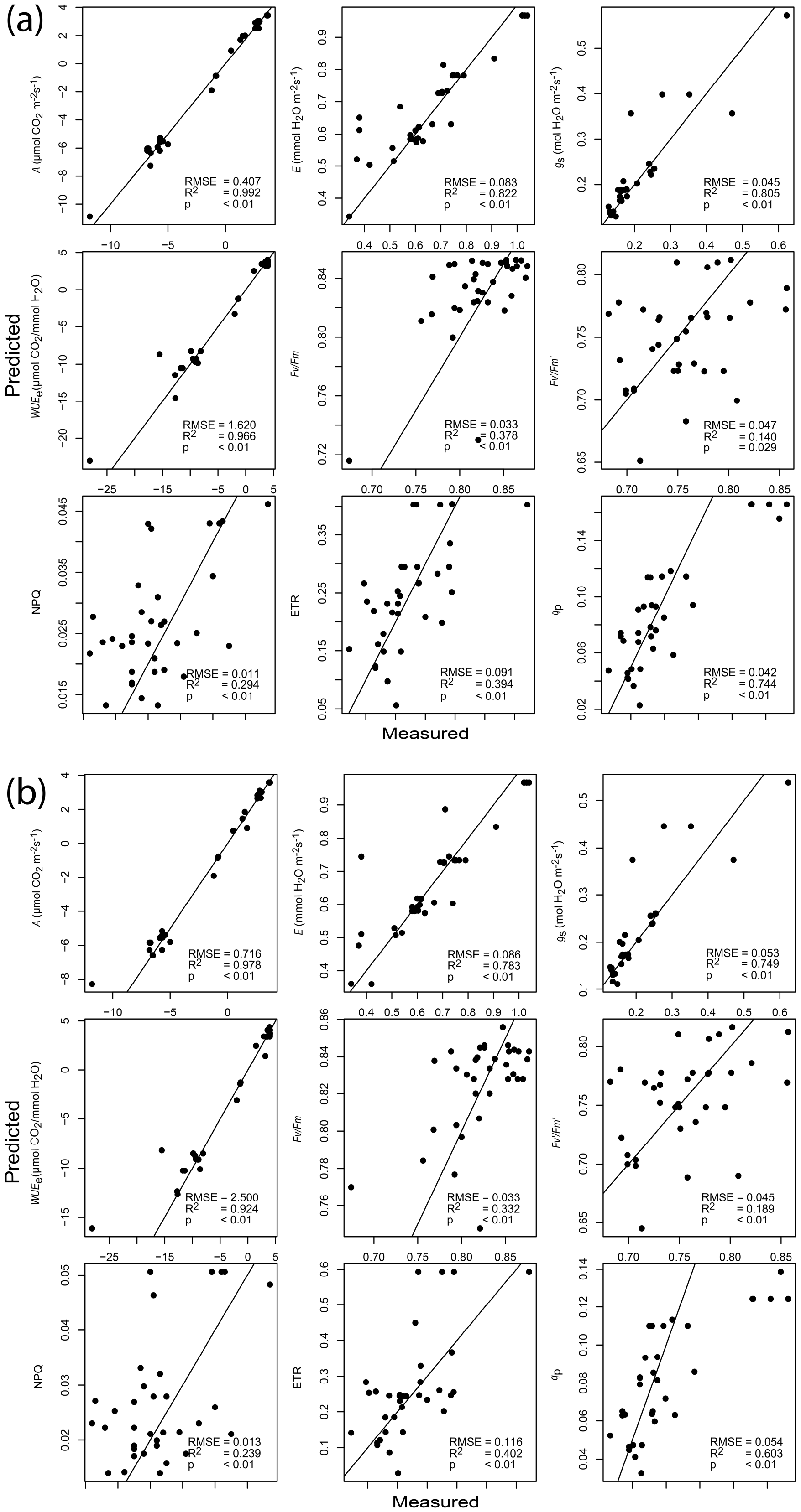
(a)
A (μmol CO2 m-2s-1)
RMSE = 0.407
R2 = 0.992
p < 0.01
E (mmol H2O m-2s-1)
RMSE = 0.083
R2 = 0.822
p < 0.01
gs (mol H2O m-2s-1)
RMSE = 0.045
R2 = 0.805
p < 0.01
Predicted
WUEe(μmol CO2/mmol H2O)
RMSE = 1.620
R2 = 0.966
p < 0.01
Fv/Fm
RMSE = 0.033
R2 = 0.378
p < 0.01
Fv'/Fm'
RMSE = 0.047
R2 = 0.140
p = 0.029
NPQ
RMSE = 0.011
R2 = 0.294
p < 0.01
ETR
RMSE = 0.091
R2 = 0.394
p < 0.01
qp
RMSE = 0.042
R2 = 0.744
p < 0.01
Measured
(b)
A (μmol CO2 m-2s-1)
RMSE = 0.716
R2 = 0.978
p < 0.01
E (mmol H2O m-2s-1)
RMSE = 0.086
R2 = 0.783
p < 0.01
gs (mol H2O m-2s-1)
RMSE = 0.053
R2 = 0.749
p < 0.01
Predicted
WUEe(μmol CO2/mmol H2O)
RMSE = 2.500
R2 = 0.924
p < 0.01
Fv/Fm
RMSE = 0.033
R2 = 0.332
p < 0.01
Fv'/Fm'
RMSE = 0.045
R2 = 0.189
p < 0.01
NPQ
RMSE = 0.013
R2 = 0.239
p < 0.01
ETR
RMSE = 0.116
R2 = 0.402
p < 0.01
qp
RMSE = 0.054
R2 = 0.603
p < 0.01
Measured

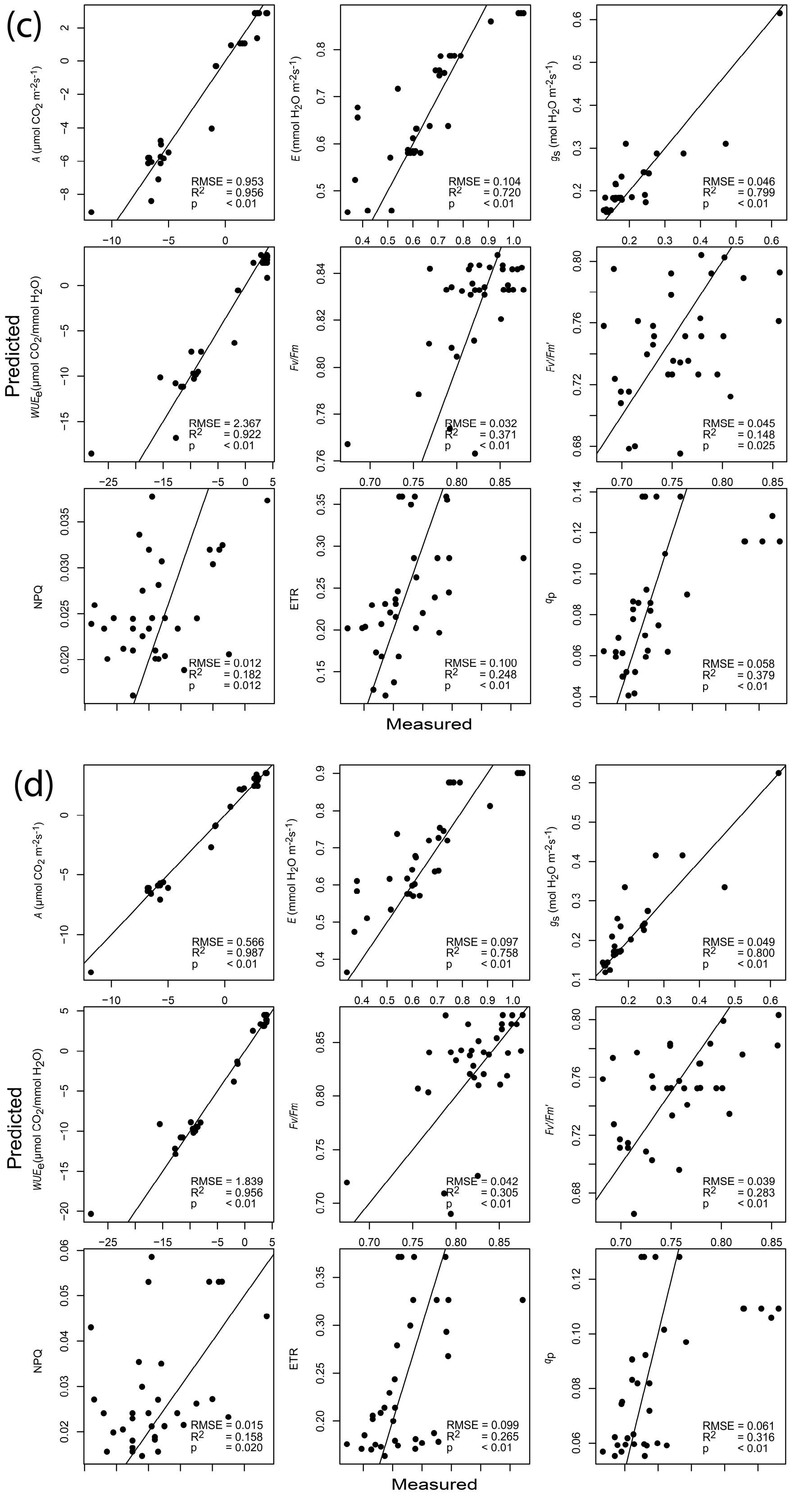

(c)
A (µmol CO2 m-2s-1)
RMSE = 0.953 R2 = 0.956 p < 0.01
E (mmol H2O m-2s-1)
RMSE = 0.104 R2 = 0.720 p < 0.01
gs (mol H2O m-2s-1)
RMSE = 0.046 R2 = 0.799 p < 0.01
Predicted
WUEe(µmol CO2/mmol H2O)
RMSE = 2.367 R2 = 0.922 p < 0.01
Fv/Fm
RMSE = 0.032 R2 = 0.371 p < 0.01
Fv'/Fm'
RMSE = 0.045 R2 = 0.148 p = 0.025
NPQ
RMSE = 0.012 R2 = 0.182 p = 0.012
ETR
RMSE = 0.100 R2 = 0.248 p < 0.01
qp
RMSE = 0.058 R2 = 0.379 p < 0.01
Measured
(d)
A (µmol CO2 m-2s-1)
RMSE = 0.566 R2 = 0.987 p < 0.01
E (mmol H2O m-2s-1)
RMSE = 0.097 R2 = 0.758 p < 0.01
gs (mol H2O m-2s-1)
RMSE = 0.049 R2 = 0.800 p < 0.01
Predicted
WUEe(µmol CO2/mmol H2O)
RMSE = 1.839 R2 = 0.956 p < 0.01
Fv/Fm
RMSE = 0.042 R2 = 0.305 p < 0.01
Fv'/Fm'
RMSE = 0.039 R2 = 0.283 p < 0.01
NPQ
RMSE = 0.015 R2 = 0.158 p = 0.020
ETR
RMSE = 0.099 R2 = 0.265 p < 0.01
qp
RMSE = 0.061 R2 = 0.316 p < 0.01
Measured

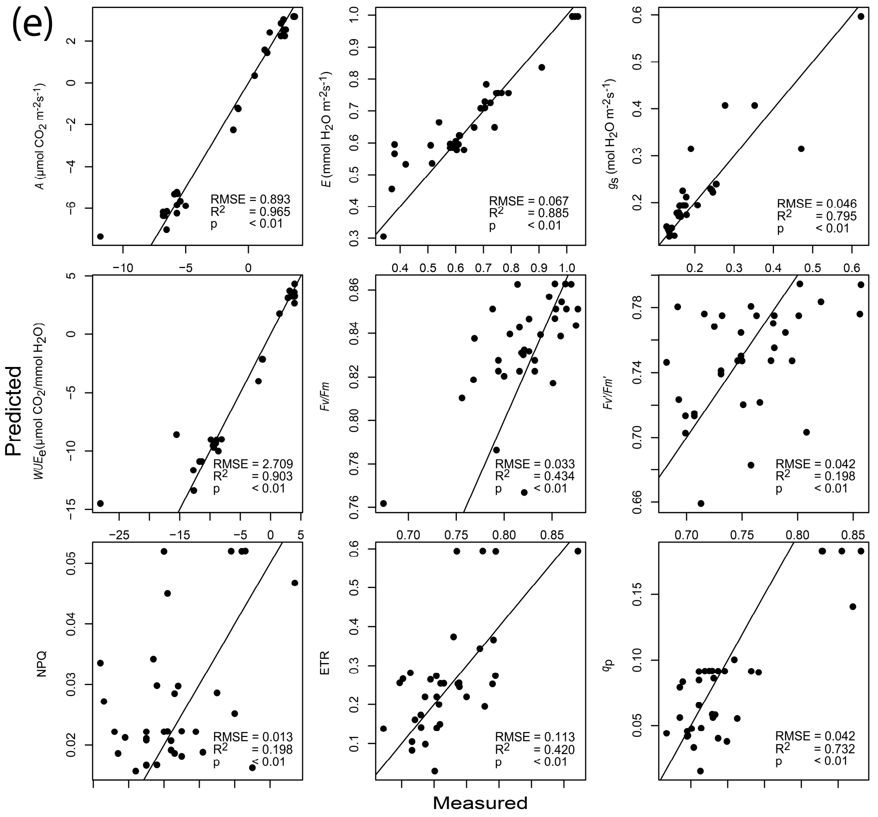

Figure S2. Predicted vs. measured values plots of the testing datasets results for five machine learning models used to predict nine photosynthetic traits: net CO_2 assimilation rate (A) (μmol CO_2 m^{-2} s^{-1}), transpiration rate (E) (mmol H_2O m^{-2} s^{-1}), stomatal conductance to water vapor (g_s) (mol H_2O m^{-2} s^{-1}), water use efficiency extrinsic (WUE_e) (μmol CO_2 mmol H_2O^{-1}), maximum quantum yield of photosystem II (PSII) (F_v/F_m), efficiency of excitation energy capture by open PSII reaction centers (F_v'/F_m'), non-photochemical quenching coefficient (NPQ), electron transport rate (ETR), and photochemical quenching coefficient (qP). (**a**) Random Forest (RF), (**b**) Boosted Regression Tree (BRT), (**c**) Bagged Classification and Regression Tree (BCART), (**d**) Artificial Neural Network (ANN), and (**e**) Support Vector Machine (SVM)
